# Molecular Signatures of the Eagle Effect Induced by the Artificial Siderophore Conjugate LP-600 in *E. coli*

**DOI:** 10.1021/acsinfecdis.2c00567

**Published:** 2023-02-10

**Authors:** Yi-Hui Lai, Raimo Franke, Lukas Pinkert, Heike Overwin, Mark Brönstrup

**Affiliations:** † Department of Chemical Biology, Helmholtz Centre for Infection Research, Inhoffenstrasse 7, 38124 Braunschweig, Germany; ‡ German Center for Infection Research (DZIF), Site Hannover-Braunschweig, 38124 Braunschweig, Germany; § Center of Biomolecular Drug Research (BMWZ), Leibniz University, 30159 Hannover, Germany

**Keywords:** *Escherichia coli*, siderophores, antibiotics, metabolomics, Eagle effect, RNA-sequencing

## Abstract

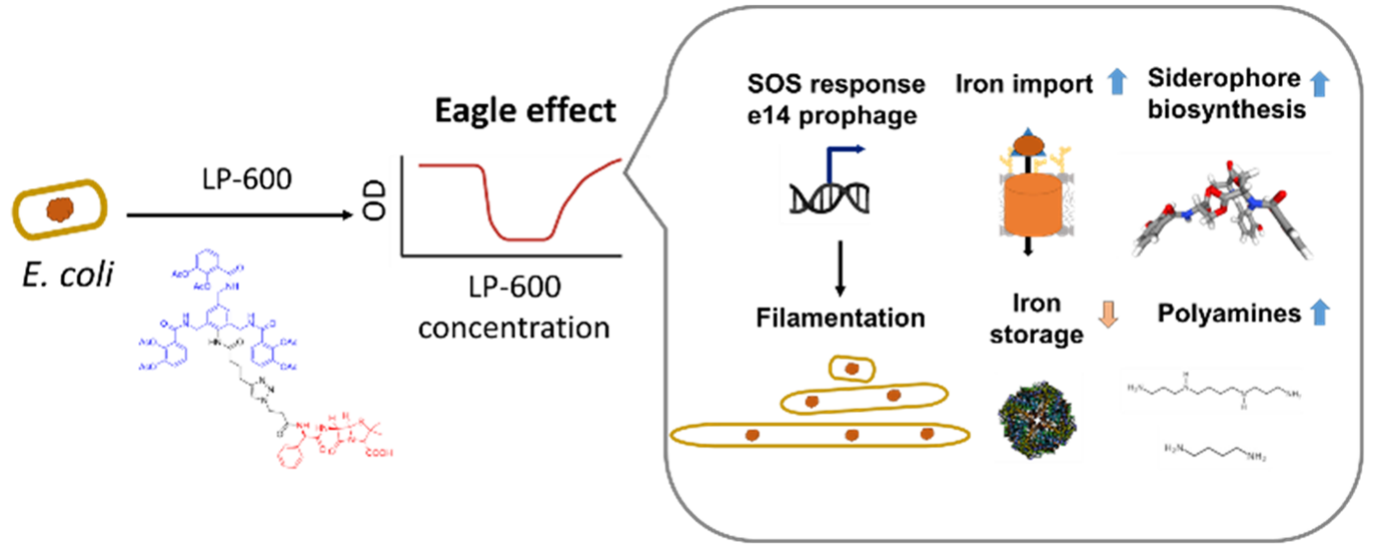

Achieving cellular uptake is a central challenge for novel antibiotics targeting Gram-negative bacterial pathogens. One strategy is to hijack the bacterial iron transport system by siderophore-antibiotic conjugates that are actively imported into the cell. This was realized with the MECAM-ampicillin conjugate LP-600 we recently reported that was highly active against *E. coli*. In the present study, we investigate a paradoxical regrowth of *E. coli* upon treatment of LP-600 at concentrations 16–32 times above the minimum inhibitory concentration (MIC). The phenomenon, coined “Eagle-effect” in other systems, was not due to resistance formation, and it occurred for the siderophore conjugate but not for free ampicillin. To investigate the molecular imprint of the Eagle effect, a combined transcriptome and untargeted metabolome analysis was conducted. LP-600 induced the expression of genes involved in iron acquisition, SOS response, and the e14 prophage upon regrowth conditions. The Eagle effect was diminished in the presence of sulbactam, which we ascribe to a putative synergistic antibiotic action but not to β-lactamase inhibition. The study highlights the relevance of the Eagle effect for siderophore conjugates. Through the first systematic –omics investigations, it also demonstrates that the Eagle effect manifests not only in a paradoxical growth but also in unique gene expression and metabolite profiles.

The World Health Organization (WHO) announced that antibiotics are becoming increasingly ineffective due to the global spread of drug resistance.^1,2^ Alarmingly, only few novel antibiotics have been recently developed that were also effective against Gram-negative pathogens.^3^ In Gram-negative bacteria, the impermeable biological barrier, imposed by an asymmetric outer membrane and a differently composed inner lipid bilayer, results in limited drug uptake.^4,5^ One strategy to facilitate drug internalization is the so-called “Trojan Horse” approach. Since bioavailable iron is often limited but essential for the survival of microorganisms, bacteria express iron chelators (“siderophores”) that capture extracellular ferric iron and then actively transport it back across the bacterial membranes.^6^ By hijacking this iron-acquisition system,^6^ siderophore-antibiotic conjugates are actively imported to the pathogen, boosting antibiotic efficacy by increasing intracellular accumulation. The most advanced Trojan Horse is the siderophore-cephalosporin conjugate cefiderocol (Fetroja), which has recently gained market approval for treating infections with carbapenem-resistant Gram-negative bacteria.^7,8^ We have recently reported that LP-600, a conjugate of the artificial tricatecholate MECAM and the β-lactam antibiotic ampicillin (Figure 1A), inhibited the growth of Gram-positive and Gram-negative multidrug-resistant pathogens at nanomolar concentrations.^9^ Compared to the bidentate monocatechol cefiderocol, the MECAM core harbors three catecholate moieties that achieve a hexacoordination of iron, thereby improving iron transportation capabilities.^9^ While the active transport of such siderophores is not operative under cultivation conditions with iron-rich media, it becomes visible when only trace amounts of iron are present, which reflects infection conditions in vivo.^10^ Furthermore, the catechols in LP-600 are masked as acetylated prodrugs to avoid in vivo deactivation of the iron-chelating units by catechol-*O*-methyltransferases.^11,12^

**Figure 1:**
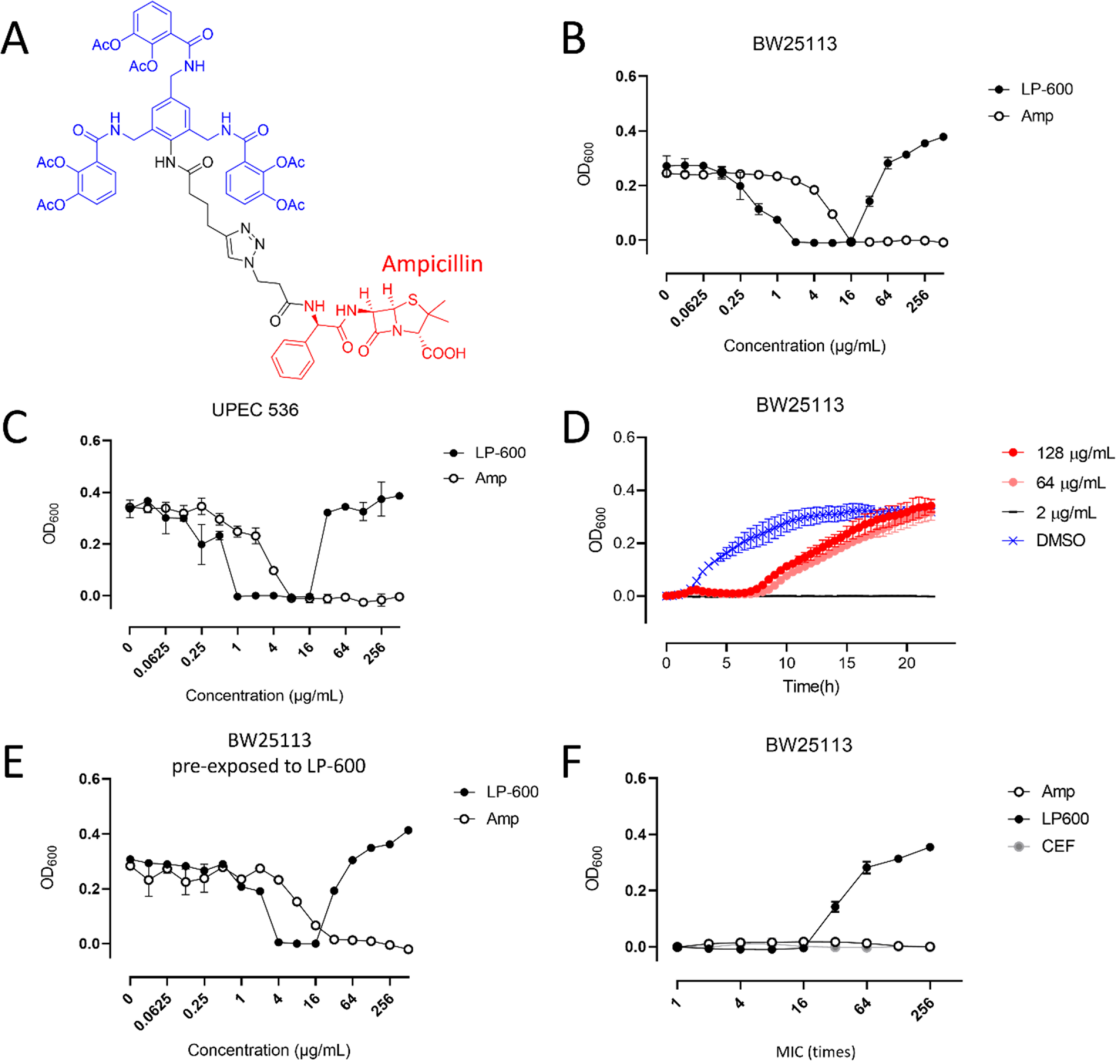
High concentrations of LP-600 induce a paradoxical regrowth of *E. coli*. (A) Chemical structure of LP-600. (B,C) LP-600-induced growth inhibition of the laboratory strain *E. coli* BW25113 (K-12) (B) and the clinical strain UPEC 536 (C) in iron-depleted Mueller Hinton broth, followed by OD_600_ measurements after 24 h. (D) Growth curves for *E. coli* BW25113 upon treatment with LP-600 or DMSO (vehicle control) followed by OD_600_ measurements over 24 h. (E) *E. coli* BW25113 cultures were treated with 128 μg/mL of LP-600 for 24 h. The cultures were harvested, washed in PBS, and used for a second growth inhibition experiment with LP-600 or ampicillin over 24 h. Concentration-dependent OD_600_ of the second experiment is depicted. (F) *E. coli* BW25113 cultures were treated at different concentrations of LP-600 (2–512 μg/mL), ampicillin (16–4096 μg/mL), or cefiderocol (0.25–64 μg/mL) over 24 h, followed by OD_600_ measurements. Amp = ampicillin, CEF = cefiderocol.

In this study, we found that exposing *Escherichia coli* to LP-600 at concentrations eight times above the minimum inhibitory concentration (MIC) resulted in a paradoxical regrowth.^10^ This phenomenon, observed before for a wide range of pathogens such as Group B *Streptococcus*, *Clostridium difficile*, *E. coli*, and diverse antibiotics (e.g., cell wall synthesis or DNA gyrase inhibitors),^13,14^ was termed “Eagle-effect” after Harry Eagle who discovered it.^15^ In addition to observations in vitro, it was reported that reduced dosage of penicillin improved treatment of bacterial infection in patients.^16^ While most studies on the Eagle effect were mainly observational, several mechanisms explaining it have been proposed,^15^ including a reduced level of reactive oxygen species (ROS)^17^ or an enhanced β-lactamase expression.^18^ Here, we demonstrate an Eagle effect that is specific for the siderophore conjugate but not for the antibiotic alone. To gain an understanding of the underlying cellular mechanisms, microbiological experiments were combined with a systemic analysis based on transcriptomics and untargeted metabolomics,^19,20^ technologies that are widely applied to study the interaction between antibiotics and microorganisms.^19−23^

## Results

### High-Concentration Treatment with LP-600 Induces a Paradoxical Growth of *E. coli*

Laboratory (K-12, BW25113) and uropathogenic (536) *E. coli* strains were sensitive to LP-600 with minimum inhibitory concentrations (MICs) of 2 μg/mL and 1 μg/mL, respectively. Surprisingly, both strains displayed a paradoxical regrowth upon treatment of LP-600 at concentrations more than 16 times higher than the MIC (Figure 1B,C). To investigate the kinetics of regrowth, the optical densities at a wavelength of 600 nm (OD_600_) were recorded over 24 h. The growth lag phase under exposure to LP-600 at high concentrations of (64 or 128 μg/mL) was prolonged from 2 h to more than 7 h compared to the growth of the vehicle control (Figure 1D). After 20 h, the high concentration treatments reached similar OD_600_ values as the vehicle control, whereas a low concentration of 2 μg/mL led to sustained growth inhibition.

In order to examine whether a culture showing the Eagle effect after 24 h of treatment with 128 μg/mL of LP-600 had become resistant, the culture was diluted and treated with LP-600 again. Bacteria from those cultures were still susceptible to LP-600 as well as to ampicillin and again exhibited the Eagle effect (Figure 1E), implying that the effect resulted from a transient phenomenon and did not reflect permanent resistance.

Goldstein and Rosdahl found that ampicillin could induce the Eagle effect in certain *E. coli* strains.^24^ To understand whether the Eagle effect observed in our experiments was also a response to β-lactam exposure at high concentrations, free ampicillin was tested. Intriguingly, *E. coli* BW25113 displayed no Eagle effect upon exposure to ampicillin at concentrations up to 256 times (4096 μg/mL) the MIC (Figure 1F). Besides, to examine whether siderophore-conjugates other than LP-600 may cause the Eagle effect in *E. coli*, the cephalosporin-monocatecholate conjugate cefiderocol was tested. No Eagle effect was observed at concentrations up to 256 times (64 μg/mL) the MIC of cefiderocol (Figure 1F). Next, the importance of iron availability was investigated. *E. coli* showed no Eagle effect following treatment with LP-600 (up to 512 μg/mL) in noniron depleted Mueller Hinton Broth (Figure S1A). However, the MIC increased from 2 μg/mL to 16 μg/mL. To assess in how far siderophore uptake may play a role in the LP-600-induced Eagle effect, *E. coli* strains lacking genes involved in siderophore transport were tested. These knockout strains were missing genes for important outer membrane receptors (*fepA, cirA,* or *fiu*), a periplasmic binding protein (*fepB*), or an import protein localized at the inner membrane (*fepD*), respectively. Yet, all strains involved in siderophore uptake displayed the Eagle effect upon exposure to LP-600 (Figure S1B,C). The effect also occurred in a knockout strain deficient of *tolC* (Figure S1C), which is involved in drug efflux and enterobactin secretion.^25^ These results are in line with previous findings that the antibiotic action of LP-600 is not impaired by single transport gene knockouts.^6^ In summary, the Eagle effect in *E. coli* was specifically induced by the MECAM-ampicillin conjugate LP-600 rather than by β-lactams or siderophore-conjugated antibiotics, in general, and it occurred in an iron-dependent manner.

### Sulbactam Diminishes the LP-600-Induced Eagle Effect in *E. coli*

**Figure 2:**
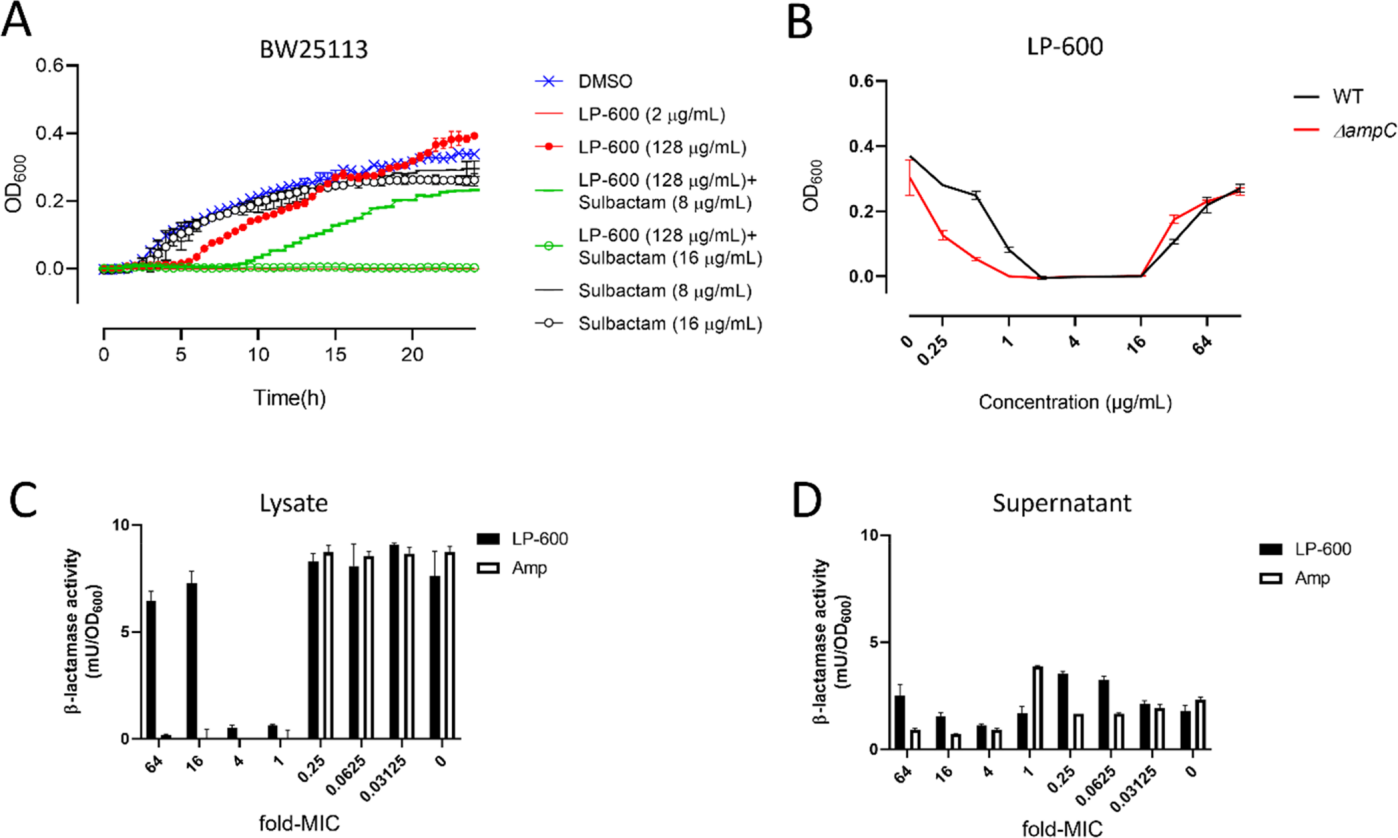
β-lactamase inhibitor sulbactam diminishes the Eagle effect induced by LP-600. (A) *E. coli* cultures were treated with LP-600 or DMSO (vehicle control), with or without sulbactam, and the growth kinetics were analyzed via OD_600_ measurements over 24 h. (B) WT and *ΔampCE. coli* strains were treated with LP-600 at indicated concentrations for 24 h in iron-depleted Mueller Hinton broth, followed by OD_600_ measurement. (C,D) *E. coli* BW25113 cultures were treated with LP-600 for 24 h, and the β-lactamase activities of bacterial lysate (C) and supernatant (D) were measured by following the absorbance (*A*_490_) of a reaction buffer containing nitrocefin, a substrate of β-lactamases. The concentrations of treatment were expressed as multiples of the MIC of LP-600 (2 μg/mL) or ampicillin (16 μg/mL).

Ikeda and Nishino found that treatment by a β-lactamase inhibitor abolished the β-lactam-induced Eagle effect at high concentration in *P. vulgaris*,^18^ indicating that the effect might be attributed to the expression of β-lactamases. To address this hypothesis, we examined a coadministration of LP-600 with the β-lactamase inhibitor sulbactam, that is widely applied in combination with ampicillin.^26^^27^ Sulbactam exhibited modest antibacterial activity against *E. coli* with an MIC of 64 μg/mL. Remarkably, cotreatment of *E. coli* with LP-600 and sulbactam (16 μg/mL) prevented any observable regrowth (Figure 2A). A sulbactam concentration of 8 μg/mL in combination with LP-600 decreased bacterial regrowth after an incubation time of 24 h and extended the lag phase in comparison with LP-600 alone.

The gene *ampC* encodes a chromosomal β-lactamase, and *ampC* mutations were found to contribute to β-lactam tolerance and resistance in clinical isolates.^28,29^ However, deletion of *ampC* in *E. coli* hardly impaired the Eagle effect upon LP-600 stimulation, even though it induced a 2-fold decrease in the MIC of LP-600 (Figure 2B). In line with this, *ampC* expression in the *E. coli* BW25113 wild type remained almost unchanged upon LP-600 treatment (128 μg/mL) compared to vehicle control or sub-MIC treatment of LP-600 (0.0625 μg/mL) (Supporting Information data set, sheet 1). We also investigated the β-lactamase activity with a colorimetric nitrocefin assay. After LP-600 treatment for 24 h, there was no significant increase in β-lactamase activity from the lysates of cells which exhibited the Eagle effect (32, 64, and 128 μg/mL, corresponding to 16-fold, 32-fold, and 64-fold the MIC, respectively) compared with those treated with sub-MICs of LP-600 (0–0.5 μg/mL) (Figure 2C). The same finding was obtained from culture supernatants (Figure 2D). Thus, we conclude that the Eagle effect cannot be ascribed to an increased action of β-lactamases like AmpC. However, the combination of LP-600 with sulbactam, a β-lactamase inhibitor with weak antibiotic activity, alleviated the effect. The two compounds exerted synergistic effects in combination, as indicated by a fractional inhibitory concentration (FIC) index of 0.4 for 8 μg/mL sulbactam and 0.5 μg/mL LP-600.

### LP-600 Induces Distinct Changes in the Transcriptome and Metabolome of *E. coli* during Exponential and Stationary Phase

**Figure 3:**
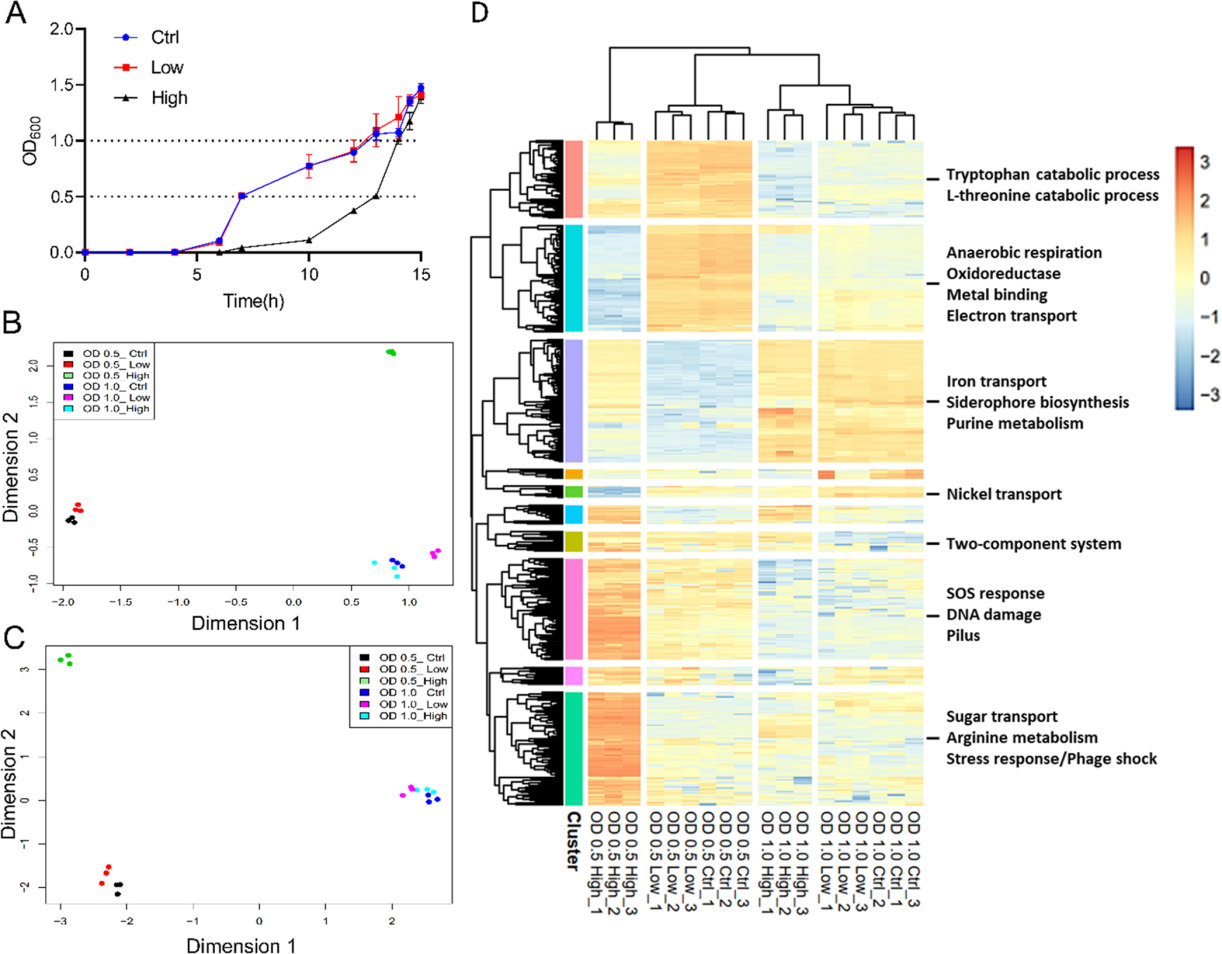
Global transcriptome and metabolome effects on *E. coli* following treatment with LP-600. (A) Growth curves of *E. coli* BW25113 treated with DMSO (control) and with low (0.0625 μg/mL; low) or high (128 μg/mL; high) concentrations of LP-600, followed by OD_600_ measurement over time. (B,C) Multidimensional scaling (MDS) analysis of *E. coli* transcriptomes obtained by RNA sequencing (B) and of metabolomes recorded by UPLC-ESI-QToF in C18 column with positive ionization mode (C) from the treatment of *E. coli* cultures with DMSO (Ctrl), or LP-600 at low or high concentrations. Cultures were harvested in the midexponential phase at OD_600_ = 0.5, or in the stationary phase at OD_600_ = 1.0. Log-counts-per-million (CPM) values from RNA-seq and log2-transformed values from metabolomics among samples were applied in MDS analysis to project Euclidean distances between samples to *x*- and *y*-axis. (D) Heat map following a hierarchical clustering of the 500 genes that had the highest variance in expression. Heatmap displays their log-CPM values with hierarchical clustering of genes and samples. Relative gene expression is color-coded from red (high expression) to blue (low expression). Ten major clusters were indicated by colored bars on the left. The heat map with dendrograms was generated by the R package limma and pheatmap. The functional enrichment analysis for each cluster of genes was conducted via STRING and DAVID WebService (version 4.0.3). The most significantly enriched terms are listed (see also the Supporting Information data set, sheet 2).

To gain a global view of the Eagle effect induced by LP-600 in *E. coli* over time, both gene expression and metabolic changes were investigated in the exponential (OD_600_ = 0.5) and stationary growth phase (OD_600_ = 1.0). Separate *E. coli* cultures were treated with a vehicle control (DMSO) or with LP-600 at low concentration (sub-MIC level, 0.0625 μg/mL) or at high concentration (128 μg/mL). Overall, six groups of samples were generated, i.e., ctrl OD_600_ = 0.5, low-concentration OD_600_ = 0.5, high-concentration OD_600_ = 0.5, ctrl OD_600_ = 1.0, low-concentration OD_600_ = 1.0, and high-concentration OD_600_ = 1.0. Because it is known that antibiotics induce cellular responses even on the sub-MIC level,^30^ the low concentration groups were introduced in order to distinguish effects induced by the mere presence of LP-600 from those that reflect the Eagle effect occurring only at high concentrations. The three samples harvested in the stationary phase had almost identical cell densities and growth times of ca. 15 h and, thus, only differed with respect to LP-600 exposure (Figure 3A). Because we hypothesized that the exponential growth phase exhibited different and relevant transcript and metabolite changes, samples at OD_600_ = 0.5 were compared with each other as well, that differed in the time points of harvesting (Figure 3A).

The transcriptomes of samples from all six groups (3 replicates each) were obtained by RNA sequencing. 4079 transcripts were annotated by mapping the obtained short reads to the genome of *E. coli* BW25113 strain (genebank: CP009273.1). To capture the overall similarity of transcriptomes, a multidimensional scaling (MDS) analysis was performed that represents the dissimilarity between objects in a data set in a two-dimensional plot (Figure 3B).^31^ In general, replicates of a given condition laid closely together, indicating a good reproducibility of the experiment. Three main clusters were clearly separated by MDS: the first one contained the three groups that were harvested at the stationary phase (OD_600_ = 1.0). The second cluster included two groups that were treated with DMSO or with a low concentration of LP-600 and harvested during the midexponential phase. The third cluster came from samples of high-concentration treatment with LP-600, harvested during the midexponential phase.

Samples for metabolomic analysis were harvested from the same batches as for transcriptome analysis, in order to enable a comparison of results from both -omics experiments. Untargeted metabolomics analysis was conducted using reversed phase and HILIC chromatography coupled to high-resolution tandem mass spectrometry in positive and negative electrospray ionization modes. For each sample, bacterial pellets were harvested, washed, and lysed to extract the endometabolites. The MDS analysis generated from metabolite feature intensities from all four metabolomics methods led to distribution patterns that were similar to the corresponding transcriptome plots (Figure 3C and Figure S2).

To display the impact of LP-600 treatments on gene expression, the top 500 most variable genes were selected by the expression variance and the heatmap displays their log-CPM values with hierarchical clustering of genes and samples (Figure 3D).^32^ The variance was calculated using the scaled log-CPM (counts per million) values of transcripts. Column-wise hierarchical clustering showed that samples harvested at OD_600_ = 1 were distinct from those harvested at OD_600_ = 0.5. The distance between samples under DMSO and low concentration treatment was small compared to those gained after high concentration exposure. Again, samples harvested at OD_600_ = 0.5 and treated with a high-concentration of LP-600 had the most distinct expression profile. These findings are in full accordance with MDS plots, and they underline that the Eagle effect was manifested in a unique gene expression profile. A row-wise hierarchical clustering was manually preselected to give ten major clusters of genes showing similar expression patterns under certain treatments. A functional enrichment analysis was conducted via the STRING tool and DAVIDWebService using Fisher’s Exact test,^33,34^ where genes are selectively classified based on GO (gene ontology) and KEGG (Kyoto Encyclopedia of Genes and Genomes) entries, and annotated in keywords by UniProt. The enriched terms were obtained through a Fisher’s exact test, and a threshold for false discovery rate (FDR) of less than 0.05 using the Benjamini-Hochberg method (Supporting Information data set, sheet 2) was set. Genes that were significantly induced upon treatment with LP-600 at high concentration in the exponential growth phase were associated with stress and SOS response, DNA damage, and phage shock (Figure 3D).

**Figure 4:**
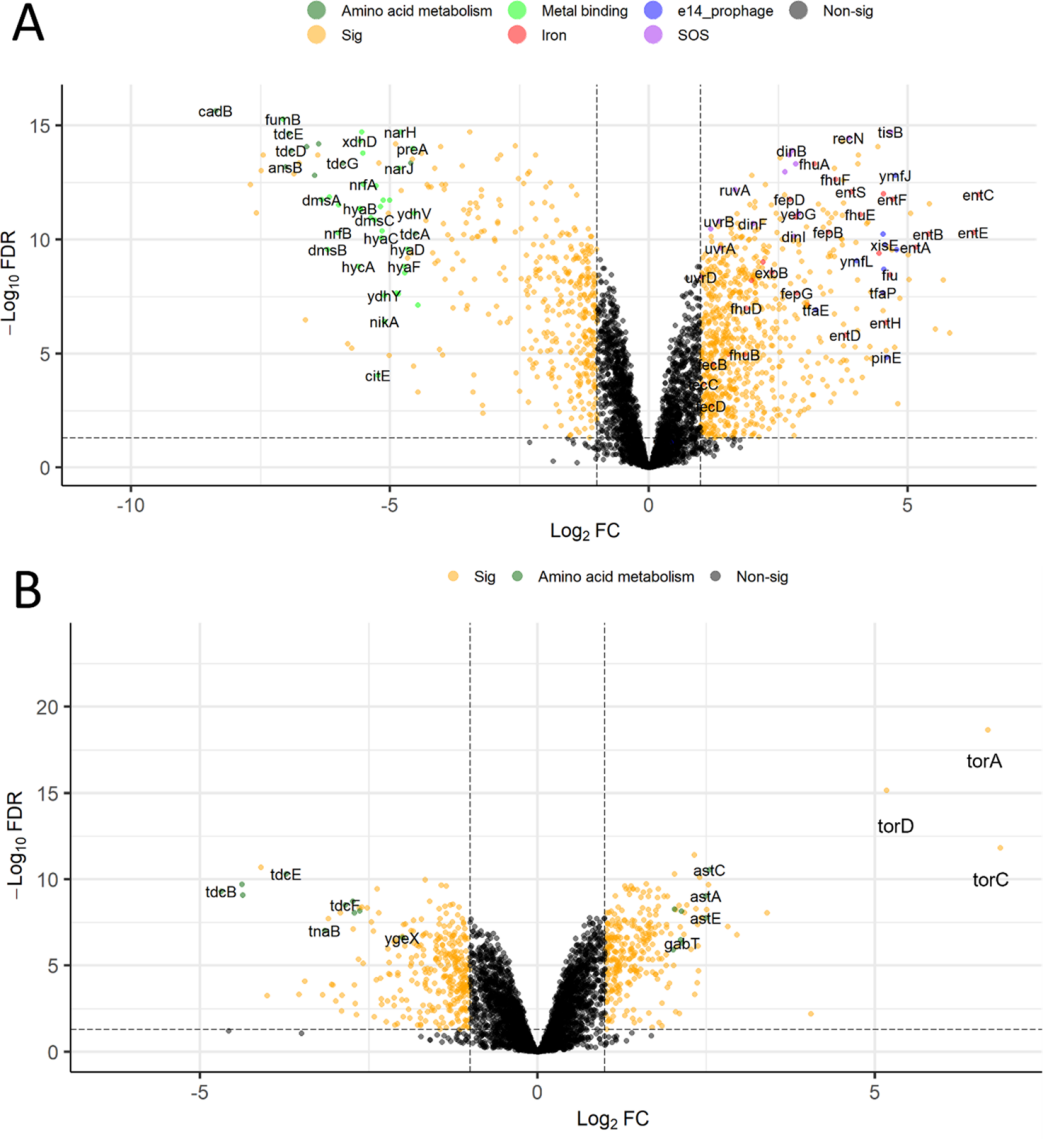
Volcano plot of differentially expressed genes from *E. coli* cultures following treatment with LP-600 at high concentrations (128 μg/mL) vs low concentrations (0.0625 μg/mL). Differentially expressed genes were obtained by subtracting the log_2_-mean values of the high concentration condition from the log_2_-mean values of the low concentration group at OD_600_ = 0.5 (LH0.5) (A) or at OD_600_ = 1 (LH1) (B). Thresholds for significantly regulated genes depicted in orange are a FDR < 0.05 and an absolute value of log_2_FC > 1. Genes belonging to enriched terms are highlighted with the indicated colors. Sig: significant expression. Nonsig: nonsignificant expression.

Of all 4079 profiled transcripts, 1182 were differentially expressed (false discovery rate < 0.05 and absolute log_2_ FC > 1) upon high-concentration treatment with LP-600 versus the low concentration treatment at an OD_600_ value of 0.5 (Figure 4A and the Supporting Information data set); we abbreviate this comparison as LH0.5. The same applied for 592 out of 4079 profiled transcripts at an OD_600_ value of 1.0 (Figure 4B and the Supporting Information data set); we abbreviate this comparison as LH1. 303 genes were shared between LH0.5 and LH1, that grouped to 22 functional categories (Figure S3 and the Supporting Information data set). Among these 22 categories, genes associated with metal-binding were represented with the largest number (71 genes). This was due to a strong impact on siderophore synthesis and transport, but also genes associated with iron utilization like iron–sulfur complexes, electron transport, and anaerobic respiration were affected (Figure S3 and the Supporting Information data set).

In the LH0.5 comparison, up-regulated genes were enriched in functional categories about SOS response, rRNA and ribosome-related pathways, and enterobactin and iron uptake, whereas iron/metal-binding and amino acid metabolism genes were down-regulated (Figure 4A, Figure S4 and the Supporting Information data set). In the LH1 comparison, genes involved in l-threonine catabolism to propionate (*tdcBCEF*) were the most down-regulated (Figure 4B, Figure S5 and the Supporting Information data set). On the other hand, genes involved in respiration or the metabolism of amino acids such as alanine, aspartate, glutamate, arginine, and proline were up-regulated. The most up-regulated genes *torACD* form an operon and encode the Tor system that was reported to carry out anaerobic trimethylamine N-oxide (TMAO) respiration in *E. coli*. It includes TorA (TMAO reductase) and TorC (a c-type cytochrome with heme-binding sites responsible for transfer electron to TorA.^35,36^

In the following paragraphs, we highlight selected processes that are considered as particularly relevant for the adaptation of *E. coli* to the siderophore conjugate.

### LP-600 Induces Iron Metabolism during the Exponential Phase

LP-600 had a profound impact on genes associated with the transport of ferric iron. Under the iron-restricted conditions of the experiments in this study, high concentrations of LP-600 in the exponential phase led to a strong (15–82-fold) up-regulation of genes involved in enterobactin synthesis (*entABCDEFHS*) (Figure 4A and Table 1). The same was true for genes coding for the outer membrane siderophore receptor FepA and the proteins FepB-G that are involved in ferric enterobactin transport from the periplasm to the cytosol. In line with this, also genes encoding the inner membrane complex TonB-ExbB-ExbD, that generates the proton motive force to enable the active transport of ferric-enterobactin by FepA, were up-regulated. Also the expression of other iron transporters, i.e., *fhuABCDEF*, *fecABCD*, cirA, and fiu, was increased in response to a high concentration of LP-600 during the exponential phase. On the other hand, genes coding for the iron storage proteins ftnA and B and iron Fe/S-cofactors hybCO were strongly down-regulated (2–42-fold) (Table 1 and the Supporting Information data set). Taken together, we observed a strong induction of the iron acquisition system in *E. coli* upon treatment with a high concentration of LP-600 during the exponential phase.

**Table 1:** Regulation of Selected Genes in *E. coli* in the Exponential Phase upon Treatment with LP-600

	gene	LH0.5_logFC	LH0.5_FDR		gene	LH0.5_logFC	LH0.5_FDR
iron transport	*entA*	5.15	2.18 × 10^–10^	LexA-regulon	*lexA*	1.99	1.54 × 10^–13^
	*entB*	5.4	5.71 × 10^–11^		*recA*	2.71	1.93 × 10^–14^
	*entC*	6.37	1.05 × 10^–12^		*sulA*	2.83	4.71 × 10^–14^
	*entE*	6.27	4.78 × 10^–11^		*tisB*	4.67	1.84 × 10^–15^
	*entF*	4.71	1.72 × 10^–12^		*recN*	3.86	3.42 × 10^–15^
	*entH*	4.57	4.27 × 10^–07^		*uvrB*	1.36	1.56 × 10^–11^
	*entS*	3.9	7.85 × 10^–13^		*sbmC*	2.63	1.07 × 10^–13^
	*fepA*	4.53	9.82 × 10^–13^		*dinI*	2.8	7.22 × 10^–11^
	*fepB*	3.47	4.68 × 10^–11^		*dinF*	2.01	2.02 × 10^–11^
	*fepC*	3.06	7.07 × 10^–08^		*uvrA*	1.37	2.34 × 10^–10^
	*fepD*	2.72	1.72 × 10^–12^		*yebG*	2.88	7.37 × 10^–12^
	*fepG*	2.85	2.21 × 10^–08^		*uvrD*	1.01	4.44 × 10^–09^
	*fhuA*	3.2	4.71 × 10^–14^		*ruvA*	1.67	6.44 × 10^–13^
	*fhuB*	1.85	1.11 × 10^–05^		*ruvB*	1.19	3.40 × 10^–11^
	*fhuC*	1.97	6.02 × 10^–09^		*dinB*	2.75	1.24 × 10^–14^
	*fhuD*	1.88	1.01 × 10^–07^				
	*fhuE*	4.08	7.90 × 10^–12^				
	*fhuF*	3.6	2.31 × 10^–13^				
	*fecA*	1.53	1.07 × 10^–05^	e14 prophage	*ymfD*	1.68	9.62 × 10^–08^
	*fecB*	1.23	2.93 × 10^–05^		*ymfE*	0.42	7.66 × 10^–02^
	*fecC*	1.06	2.14 × 10^–04^		*ymfI*	1.82	1.01 × 10^–06^
	*fecD*	1.2	1.95 × 10^–03^		*ymfJ*	4.76	1.54 × 10^–13^
	*fiu*	4.66	3.39 × 10^–09^		*ymfL*	4.01	8.38 × 10^–10^
	*cirA*	4.44	3.92 × 10^–10^		*ymfM*	4.54	1.94 × 10^–09^
	*tonB*	2.2	9.67 × 10^–10^		*ymfQ*	4.78	2.69 × 10^–10^
	*exbB*	2.37	2.67 × 10^–09^		*ymfR*	4.52	5.78 × 10^–11^
	*exbD*	2.84	1.01 × 10^–11^		*lit*	2.26	1.11 × 10^–08^
	*hybA*	–5.38	1.07 × 10^–11^		*pinE*	4.59	1.44 × 10^–05^
	*hybO*	–5.19	3.57 × 10^–12^		*tfaE*	3.23	1.20 × 10^–07^
	*ftnB*	–0.97	9.36 × 10^–05^		*tfaP*	4.52	2.10 × 10^–08^
	*ftnA*	–3.53	1.84 × 10^–11^		*xisE*	4.56	1.69 × 10^–10^

### LP-600 Induces the SOS Response during Exponential Phase

A cluster of genes belonging to the *lexA* regulon was significantly induced by LP-600 during the midexponential phase (LH0.5) (Figure 4A, Table 1, and Supporting Information data set). The *lexA* regulon is part of the SOS response, a global reaction to DNA damage or antibiotic treatment, that was found to be associated with the evolution of resistance under treatment with antibiotics such as β-lactams and DNA-damaging fluoroquinolones in previous studies.^37−39^ Stress or DNA damage activates the transcription factor RecA to stimulate self-cleavage of LexA, leading to the expression of SOS genes for repair.^38^ It has been shown before that β-lactam-induced cell wall stress led to the activation of the DpiBA two component signal transduction system, resulting in SOS induction.^40^ Iron depletion due to complexation with LP-600, but an insufficient intracellular iron release, could provide an alternative explanation for SOS induction. Leaden et al. showed that lexA and most of its target genes were up-regulated in the Gram-negative bacterium *C. crescentus* in response to iron depletion in the growth medium by the iron chelator 2–2-dypiridyl.^41^ Transcriptional up-regulation of the *sulA* gene, also observed by us (Table 1), can then lead to the blocking of the FtsZ ring formation by SulA-binding, preventing cell division and providing protection against cell death.^40^

For the SOS gene *tisB*, up-regulated 25-fold, Dörr et al. showed that its overexpression significantly induced the formation of persister cells.^42^ The authors also revealed that TisB-dependent persisters were highly tolerant to many antibiotics, indicating that SOS response and TisB provided survival benefits for bacteria under antibiotic exposure by disrupting the proton-motive force PMF and interfering with antibiotic uptake. Because the uptake of LP-600 is strictly dependent on TonB-coupled outer membrane transporters, that are driven by the PMF, the TisB increase may restrict uptake of the antibiotic conjugate over time.

### LP-600 Induces the Expression of the e14 Prophage Region during Exponential Phase

Thirteen genes that belong to the e14 prophage were differentially up-regulated by 1.3–27-fold in the exponential phase (Figure 4A and Table 1). The e14 element is one of the defective prophages integrated into the *E. coli* chromosome,^43,28^ and most genes encode proteins with unknown functions. However, a few were found to have an association with inhibition of cell division and filamentation.^44,45^ Ansari et al. showed that overexpression of the SOS-induced *ymfM* gene caused filamentation and inhibition of Z ring formation, thereby halting cell division.^45^ Filamentation helps *E. coli* to survive in different environments and contributes to antibiotic resistance.^45^ Miller et al. reported that β-lactam-induced SOS response and filamentation enabled bacteria to survive upon exposure to antibiotics by reducing the necessity for cell wall synthesis.^37^ In addition to the upregulation of ymfM, the inhibition of PBP3 itself by ampicillin could also lead to filamentation as reported before.^46,47^

**Figure 5:**
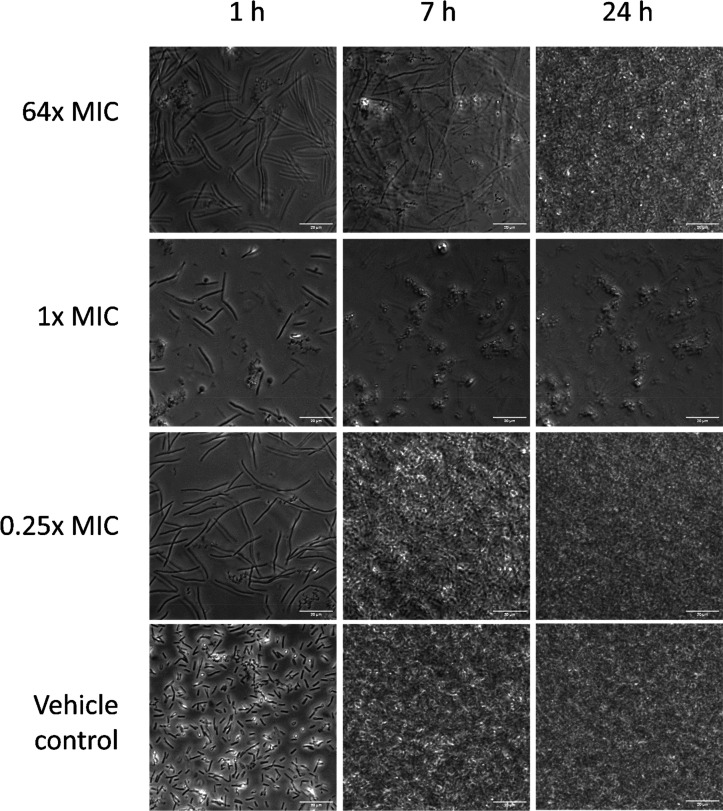
Micrographs of *E. coli* BW25113 treated with LP-600 or vehicle control. *E. coli* BW25113 was treated with LP-600 at 64× MIC (128 μg/mL), 1× MIC (2 μg/mL), 0.25× MIC (0.5 μg/mL), and vehicle control at 37 °C in iron-depleted Mueller Hinton broth, followed by time-lapse microscopic measurements under 100× oil immersion of phase-contrast view. Scale bars: 20 μm.

In order to verify whether filamentation also occurred under LP-600 treatment, the bacterial growth morphology was investigated by time-lapse microscopic imaging. While cell growth and proliferation were inhibited by LP-600 at one-fold MIC, growth occurred both at 0.25-fold MIC as well as at 64-fold MIC and also in the vehicle control (Figure 5). This finding is in line with the growth kinetics determined by OD measurements (Figure 1D). An elongation of bacterial cells compared to the vehicle control was clearly visible at 0.25-fold MIC and at 64-fold MIC after 1 h of incubation. After 7 h of incubation, elongated cells were still present at 64-fold MIC, but the cell number was lower compared to the treatment at 0.25-fold MIC. This reflected the increase of the lag phase at high concentrations (Figure 1D). After 24 h, the density of the cell layers at 0.25-fold MIC and at 64-fold MIC was comparable to that of the vehicle control. In contrast, few cells were visible at one-fold MIC.

The observed cell phenotypes led to the hypothesis that the increase in OD at high concentrations of LP-600 might not be a result of cell proliferation, but of the pronounced elongation of a smaller number of cells. To probe this, we determined the number of colony forming units (CFUs) after 20 h of incubation with LP-600. The Pearson coefficient for the correlation between OD_600_ and CFU counts is 0.87 (Table S5). This illustrates that the optical densities even under conditions of the Eagle effect reflected cell proliferation and were not strongly impacted by cell elongation.

### Metabolomic Changes Following Treatment with LP-600

To identify individual small molecule metabolites that marked the Eagle effect, the abundance of individual features from the high concentration treatment with LP-600 was compared to the respective low concentration group for both growth phases. Overall, 179 metabolites could be identified by matching their high-resolution masses, retention times, and tandem MS spectra to internal and external reference databases (Supporting Information data set). The majority of them (122 metabolites, 68%) were amino acids or peptides. Metabolites were considered as significantly regulated if they had an absolute fold change in abundance of >1.5 and a FDR < 0.05. These criteria were met by 120 metabolites only in LH0.5 (96 increased and 24 decreased) and by 19 metabolites only in LH1 (12 metabolites increased and 7 decreased). 45 metabolites were significantly changed in both conditions: 19 were increased in both conditions, one was decreased in both conditions, 7 were decreased in LH0.5 and increased in LH1, and 18 were increased in LH0.5 and decreased in LH1 (Supporting Information data set). The stronger differential regulation of the metabolomes in the LH0.5 comparison versus LH1 is in line with the transcriptome effects that were also more pronounced for LH0.5 (see above). Because the biochemical interpretation of the effects was hampered by the incomplete annotation of the metabolomes, only a few observations are highlighted.

Riboflavin was increased in the LH0.5 comparison. Iron physiological status influences riboflavin biosynthesis, mediated by iron-riboflavin regulatory interplay.^48^ The transformation of a siderophore-deficient *E. coli* strain with ribBA from *H. pylori* has been shown to restore growth in a low iron environment.^48,49^ Therefore, we speculate that riboflavin biosynthesis was increased to overcome the iron limitation that was prevalent in our experiments.

In the exponential growth phase, metabolites of increased abundance included basic amino acids and polyamines such as spermidine,^21^ N^1^-acetylspermidine (2^0.63^), N^8^-acetylspermidine (2^0.62^), cadaverine (2^1.17^), or agmatine (2^3.03^). This overexpression was weaker or disappeared in the stationary phase. Polyamines modulate diverse cellular functions such as ribosomal activities, permeability, and protection from oxidative stress. The increase in polyamines such as cadaverine and spermidine upon exposure to LP-600 are in line with Tkachenko et al.’s finding of polyamine induction upon a sub-MIC treatment with β-lactams.^50^ It was also reported that polyamines protect DNA and proteins from oxidative stress,^51^ thereby enhancing antibiotic resistance and survival.^52^ On the other hand, Kwon and Lu showed that the exogenous addition of polyamines decreased the MIC of ampicillin and other β-lactams in *E. coli*;^53^ given the applied high, millimolar concentrations, this might be a consequence of membrane effects. In order to test whether exogenous polyamines are involved in the Eagle effect of LP-600, the growth inhibition experiment shown in Figure 1B was repeated in the presence of 1 mM spermidine and cadaverine (Figure S6). We observed that the addition of exogenous polyamine did not change bacterial growth. However, this does not exclude that the increased polyamine expression is important for the intracellular response to antibiotic exposure.

The level of oxidative stress is also indicated by the ratio of reduced glutathione (GSH) to its oxidized form GSSG.^54^ GSH is an important scavenger of reactive oxygen species (ROS)^54^ and decreased when confronted with more ROS, while the concentration of the oxidized form GSSG increases. During the exponential phase (LH0.5), GSH was increased (2^0.89^) and GSSG decreased (2^–1.94^). These findings were reversed during the stationary phase, where GSH was strongly decreased (2^–7.15^) and GSSG increased (2^1.33^) when comparing high versus low concentration of LP-600. Whether the higher GSH/GSSG ratio reflected efficient radical scavenging by polyamines,^51^ or a generally reduced oxidative stress environment cannot be concluded from the metabolome data.

## Discussion

The current study highlights that the Eagle effect, reported for a variety of nonconjugated antibiotics,^55^ was also operative in a siderophore-antibiotic conjugate. The effect occurred in an iron-dependent manner, and it was specific for the conjugate, not for the antibiotic alone. Although we demonstrated that regrowth was not due to genetic resistance formation, we probed whether the effect could be traced back to a transient overexpression of β-lactamases, which constitute the major inactivation mechanism for β-lactams in *E. coli*. In fact, Ikeda et al. reported that the presence of β-lactamase inhibitors or β-lactamase-deficient strains abolished the Eagle effect induced by β-lactams (such as cephalosporins) in *P. vulgaris*.^18^ In line with this, cotreatment with sulbactam abolished the Eagle effect induced by LP-600 in *E. coli* (Figure 2). However, there was no increase in biochemical β-lactamase activity (Figure 2C,D) under LH1 conditions because *ampC*, the main chromosomal gene encoding β-lactamase, was not significantly up-regulated.^28,29^ While sulbactam is one of the most frequently used β-lactamase inhibitors, it is not devoid of antibiotic activity. For example, it is the active antibiotic component, inhibiting PBP1 and PBP3, in a novel combination therapy for the treatment of carbapenemase-resistant *Acinetobacter baumannii* infections.^56^ Notably, a synergistic effect of sulbactam and ampicillin has been observed previously in strains with low β-lactamase activity.^57^ Although the inhibitory activity of sulbactam against *E. coli* was weak,^58^ we hypothesize that the restoration of efficacy in our combination experiments might be due to an antibiotic synergism,^59^ amplified by LP-600-induced processes discussed in the following text.

The Eagle effect, although known for more than 70 years, has been hardly characterized on a molecular level by a systematic -omics study so far.^55^ In order to learn about the molecular underpinning of the cellular phenotypes, we conducted a combined transcriptome and metabolome study from the same samples. The overall clustering of samples was equivalent on both the transcript as well as the metabolite level. Hierarchical clustering as well as MDS plots demonstrated that the differences between low and high concentration treatments was much larger in the exponential growth phase compared to the stationary phase. The effect of a sub-MIC treatment compared to untreated controls was discernible in the transcriptome (Figure 3D), but relatively small overall. Beyond their benefit for group recognition on the overall feature level, the analysis of metabolomes was restricted to few, single observations because an incomplete metabolite coverage, a key technological limitation in particular for bacterial metabolomes, hampered conclusions on how full metabolic pathways were affected.

The analysis of 4079 transcripts disclosed a strong induction of the iron acquisition system in *E. coli* upon treatment with LP-600. This included the upregulation of multiple siderophore transport genes; on the other hand, iron storage genes were downregulated. We have recently highlighted the relevance of the transporter-coding genes *fepA*, *cirA*, and *fiu* for the import and efficacy of LP-600 because a triple knockout of them conferred resistance to the antibiotic. FepA was found to be the major transporter of the MECAM core in *E. coli*.^9^ Thus, the up-regulation of such transporters in the presence of large amounts of siderophore under iron-restricted conditions reflected a response directed toward satisfying iron demand. These data are also congruent with proteomic data from a study of LP-600 in *P. aeruginosa*.^60^ Here, the import of the unconjugated MECAM core also induced the expression of the catechol-type outer membrane transporters PfeA and PirA, whereas LP-600 led to an increase in the expression of *pfeA* and *ampC*, the gene conferring ampicillin resistance. Interestingly, the expression of genes encoding the endogenous siderophores pyochelin and pyoverdine was repressed in the presence of the artificial siderophores in *P. aeruginosa*, whereas siderophore biosynthesis genes were strongly upregulated in this study. This opposite trend might be due to the different concentration regimens. *P. aeruginosa* was exposed to 10 μM of LP-600, whereas 128 μg/mL (92 μM) were applied here. Although the ability of MECAM to deliver iron into *E. coli* has been proven,^9^ the large excess of the siderophore may lead to iron starvation in the (iron-depleted) growth medium and, in consequence, to an upregulation of transporters as well as siderophore synthesis. Alternatively, the upregulation of the siderophore biosynthesis pathway might actually be part of an oxidative stress response. It has been shown that enterobactin plays a role in protecting against oxidative stress. Peralta et al. observed that strains impaired in enterobactin production were more susceptible to oxidative damage than the wild-type strain.^61^ The authors hypothesized once iron is released in the cytoplasm, the free hydroxyl groups of enterobactin can be used for radical stabilization. Another more recent study observed that the Fur-regulated biosynthesis pathway was the second most up-regulated pathway after addition of a sublethal concentration of hydrogen peroxide.^62^

The Eagle effect was associated with a pronounced SOS response. Previous studies suggested that SOS response, a global response to DNA damage or antibiotics treatment, is associated with the evolution of resistance under treatment with antibiotics such as β-lactams and DNA-damaging fluoroquinolones.^37,38^ Dörr et al. showed that overexpressing *tisB*, one of the SOS genes, significantly increased the level of persister cells.^42^ They also revealed that TisB-dependent persisters were highly tolerant to many antibiotics, indicating that SOS response and TisB provide survival benefits for bacteria under antibiotic exposure by altering the PMF and interfering with antibiotic uptake. In line with their findings, a cluster of genes involved in SOS response belonging to the LexA regulon (such as *tisB*, *dinB*, *cho*, *umuC*, *recN*, etc.) were significantly induced in samples showing the Eagle effect during the midexponential phase (LH0.5) in this study (Figure 4A and Table 1). Additionally, the induction of e14 prophage genes might contribute to the Eagle effect upon SOS response; in particular, the SOS-induced *ymfM* gene caused filamentation and inhibition of cell division.^44,45^ In line with their findings, we also observed a prolonged filamentation phenotype upon LP-600 treatment (Figure 5). However, after prolonged exposure, the cell number was not reduced, as proven by CFU counts.

With regard to the translational potential of LP-600, the Eagle effect might not be relevant. Although bell-shaped concentration–response curves raise principal concerns because an efficacy window might have an upper limit. However, the successful clinical use of many antibiotics against pathogens that display an Eagle effect in cell culture is proven, probably because the elevated concentrations required for regrowth are hard to sustain in vivo.^55^

**Figure 6:**
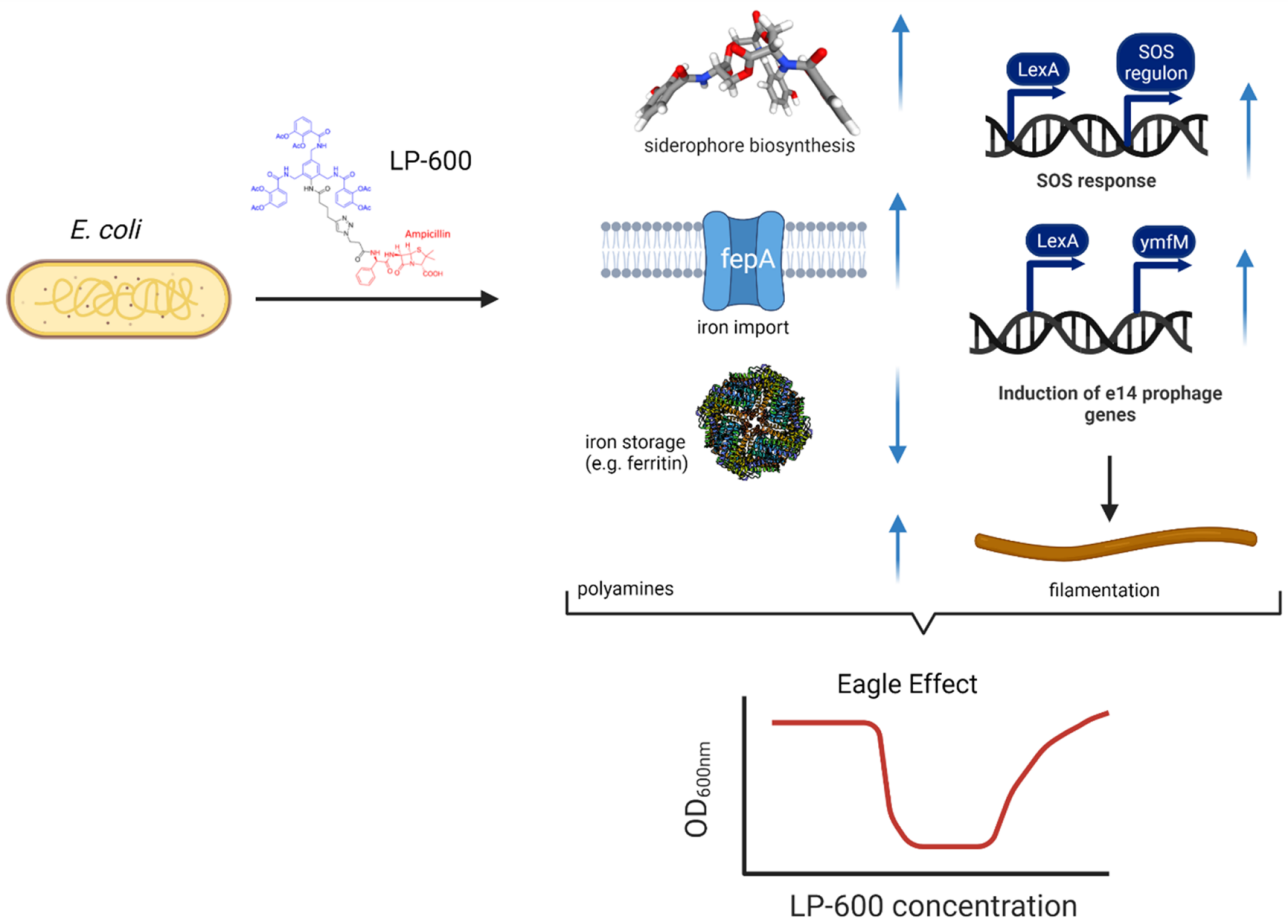
Overview on cellular processes associated with the Eagle effect upon exposure of *E. coli* to LP-600. Blue arrows indicate up or downregulation. Created with BioRender.com.

In summary, we demonstrate that also siderophore-conjugated antibiotics might be prone to the Eagle effect. By a combination of microbiological experiments with a systematic multiomics-study, we demonstrate that the effect is iron-dependent and imply that SOS response and e14 prophage genes, the iron response, and polyamine expression were major hallmarks of it (Figure 6). The mechanistic understanding of the microbial response to siderophore-coupled antibiotics may help in establishing the conjugates as a viable antibiotic molecular format. Some of the molecular mechanisms described might also occur for other compounds that were described to induce an Eagle effect, but they cannot simply be generalized. The molecular mechanisms causing the Eagle effect are under-investigated, and more -omics studies on different compounds of a given class, but also on different strains of a given bacterial species, are needed to understand the heterogeneity of responses.

## Methods

### Strains

The Keio collection strain of *E. coli*, wild-type strain BW25113, and genetically modified strains were purchased from Horizon (Cambridge, UK). In this study, several deletion strains listed below were generated by the lambda red recombinase system (Table 2).^63^

**Table 2:** Deletion Strains Generated by the Lambda Red Recombinase System

strain	reference or source	strains	reference or source
BW25113	Horizon cat. no. OEC5042	*ΔcirA*	this study
*ΔfepA*	this study	*Δfiu*	this study
*ΔfepB*	this study	*ΔampC*	Horizon cat. no. OEC4987–200828975
*ΔfepD*	this study	*ΔtolC*	Horizon cat. no. OEC4987–213607439

### Recombination/Lambda Red-Mediated Gene Deletion

Gene deletion was conducted via lambda red recombination in *E. coli* wild-type BW25113^63^ or indicated strains. Targeted genes on the corresponding locus were replaced by homologous recombination with the chloramphenicol cassette including an FRT sequence which is flanked by 50 base pairs upstream and downstream of the targeted gene. The chloramphenicol cassette was amplified from pKD3 by PCR with the primers containing corresponding upstream and downstream of the gene sequence. The primers are listed in Table S3. For expression of the lambda red system, the plasmid pkD46, encoding lambda red genes with the araBAD promoter, was transformed into the target strain and grown at 30 °C. Strains with pKD46 were further grown until the early exponential phase (OD_600_ = 0.2), followed by the induction of “lambda red” proteins Gam, Exo, and Beta by supplementation with 0.2% arabinose. Bacterial cultures were grown up to an OD_600_ value of 0.6 and transformed with the PCR product. The transformed cells were incubated for 2 h at 37 °C and then selected on an agar plate with chloramphenicol (25 μg/mL). Indicated gene deletion strains were obtained and checked by colony PCR with the primers listed in Table S4.

### Determination of Minimum Inhibitory Concentrations (MICs)

For iron-depleted Mueller Hinton Broth (MHB) preparation, MHB was treated with Chelex (Bio-Rad cat. no. 142-2842, Hercules, CA, USA) for two hours at room temperature, followed by filtration with a 0.2-μm filter, supplemented with ZnSO_4_ = 10 μM, CaCl_2_ = 0.5 mM, and MgSO_4_ = 0.5 mM, and an adjusted pH to 7.4.^64^ Serial 2-fold dilution series of tested antibiotics were prepared and dispensed in half-area 96-well plates (Greiner cat. no. 675161). Overnight cultures of each strain were diluted into OD_600_ = 0.1 and harvested when reaching the exponential phase (OD_600_ = 0.5). Bacterial pellets were harvested, washed three times in PBS, diluted into an OD_600_ value of 0.01 in iron-depleted MHB medium, and then transferred to wells with dispensed antibiotic solution in half-area 96-well plates. The plates were incubated for 24 h at 37 °C, followed by an OD_600_ measurement in a plate-spectrophotometer as mentioned above.

### Determination of CFUs from the MIC Assay

The MIC determination assay was carried out as described above. 100 μL of bacterial suspension were transferred to Eppendorf tubes from a well of the MIC test plate. After centrifugation, the supernatant was removed, and the pellet was washed three times with PBS and resuspended in 100 μL PBS. 90 μL of the suspension were diluted with 810 μL PBS. These bacterial suspensions were further diluted with PBS to reach the target dilution. From this final suspension, 10 μL (4 replicates per well) were transferred to an MHA-plate and incubated for 20 h at 37 °C and 70% humidity in an incubator without shaking. The CFU counting was carried out by eye.

### Determination of β-Lactamase Activity

The β-lactamase activity was determined by a β-lactamase activity assay kit (Sigma cat. no. MAK22) following the manufacturer’s instruction. Culture supernatants and pellets were harvested. Bacterial pellets were lysed by sonication for 5 min on ice, followed by centrifugation at 12,000*g* for five minutes, and the cell lysate was collected. Culture supernatant and cell lysate were dispensed into a 96-well plate and mixed with a reaction buffer containing nitrocefin, which is a substrate of β-lactamases. The absorbance (*A*_490_) of each well was measured with the microplate reader (BioTek Synergy5) in kinetic mode for two hours at room temperature.

### RNA Isolation and Transcriptome Analysis

Overnight cultures of each strain were diluted into OD_600_ = 0.1 and harvested until reaching the exponential phase (OD_600_ = 0.5). Bacterial pellets were harvested, washed three times in PBS, and diluted (OD_600_ = 0.01) in iron-depleted MHB medium. Two mL of *E. coli* BW25113 were treated with LP-600 at the concentration of 0.0625, 128 μg/mL or DMSO as vehicle control. Bacterial cultures were grown and pelleted by centrifugation at OD_600_ values of 0.5 and 1.0, respectively. RNA was extracted by applying the RNAeasy Mini Kit (Qiagen cat. no. 74104) and followed by the DNase treatment (Qiagen cat. no. 79254). Total RNA qualities and integrities were assessed using the 5200 Fragment Analyzer System (Agilent Technologies). By applying the NEBNext Ultra II Directional RNA Library Prep Kit (New England BioLabs), a total RNA library was generated from 1 μg total RNA after rRNA depletion using the Ribo-off rRNA Depletion Kit (Bacteria) (Vazyme BioTech Co.Ltd.). RNA-seq was performed by the Genome Analytics Research Group at the Helmholtz Center for Infection Research. The libraries were sequenced on an Illumina NovaSeq 6000 with an average of 10 × 10^6^ reads per RNA sample, using the NovaSeq 6000 S1 PE Reagent Kit (100 cycles). The sequencing image data was transformed into raw reads and saved in FASTQ format. Quality control and adapter clipping were done using the fastq-mcf tool (version: 1.04.803) of ea-utils (version:1.1.2-806 ),^65^ followed by mapping to the genome of *E. coli* strain BW25113 (genebank: CP009273.1) via the Rockhopper tool.^66^ The raw counts of reads were normalized, and differential expression analyses were performed with the R package-limma (version 3.42.2).^67^*t* test correction was obtained by false discovery rate correction through the Benjamini-Hochberg method.^68^ The cutoffs of differentially expressed genes are an absolute log 2 fold change of 1 and a corrected *p*-value of 0.05.

### Metabolomics Sample Preparation

Wild-type *E. coli* cultures were prepared as mentioned above, treated without or with LP-600 at indicated concentrations, and grown until OD_600_ = 0.5 and 1.0, respectively. For intrametabolome extraction, bacterial cultures were pelleted by centrifugation at 9000*g* for 10 min at 4 °C, followed by a wash with ice-cold aqueous NaCl solution (0.9%) and transfer into 2-mL Eppendorf tubes. The pellets were submitted to shock-freezing in liquid nitrogen and thawed three times at room temperature, respectively. The pellets were resuspended in 600 μL of ice-cold methanol spiked with glipizide as an internal standard at a concentration of 1200 μg/mL. The suspension was frozen in liquid nitrogen again, thawed, and 600 μL of ddH_2_O were added, followed by two additional freeze–thaw–sonication cycles. Extracts were centrifuged for 10 min at 13,000*g*. The supernatant was collected into fresh Eppendorf tubes and dried overnight in a speedvac. The dried metabolite extracts were reconstituted in 45 μL of 1:1 methanol/H_2_O containing trimethoprim (Sigma-Aldrich cat. no. T7883) at a concentration of 200 μg/mL and nortriptyline (Sigma-Aldrich) at a concentration of 200 μg/mL as internal controls for normalization.

### LC–MS/MS Measurement and Data Analysis

To analyze metabolomics samples, 3 μL of metabolome extracts per sample were analyzed by HR-LCMS. For LC separation, an Ultimate 3000 UHPLC-system (Dionex/Thermo Scientific, Dreieich, Germany) and a maxis HD UHR-TOF mass spectrometer (Bruker, Bremen, Germany) equipped with an Apollo II electrospray source for measuring the HR-mass data were used. Full scans (50–1500 Da) were performed for electrospray ionization (ESI) at a scan rate of 10 Hz. To generate ions, a capillary voltage of 4500 V, nebulizer pressure of 4.0 bar, dry heater of 200 °C, and a dry gas flow of 9.0/min were applied. The separation was done with a Kinetex 1.7 μm C18 150 × 2.1 mm diameter column (Phenomenex, USA) at a flow rate of 300 μL/min. Samples were analyzed in both, positive and negative modes with solvent A (water + 0.1% formic acid) and solvent B (acetonitrile + 0.1% formic acid). The elution was run as follows: 1% B from 0 to 2 min, a linear gradient from 1% to 100% B for 2 to 20 min, 100% B from 20 to 25 min, and the gradient from 100% to 1% B from 25 to 30 min. To analyze more polar metabolites, a hydrophilic interaction liquid chromatography (HILIC) separation mode was used. An Acquity UPLC BEH Amide 1.7 μm, 150 × 2.1 mm column (Waters Corp., USA) at a flow rate of 300 μL/min was applied for separation. The MS parameters were the same as for the C18 chromatography. Samples were analyzed in both, positive and negative ion mode with solvent A (Water with 20 mM ammonium formate) and solvent B (95% acetonitrile, 5% water with 20 mM ammonium formate). The elution was run as follows: 100% B from 0 to 2 min, a linear gradient from 100% to 50% B from 2 to 20 min, 50% B from 24 min, and afterward returning to 100% B. For internal calibration, sodium formate was infused into the system as a calibrant during the first 0.3 min of each run. For lock mass calibration, hexakis (2,2-difluoroethoxy) phosphazene was used with 622.0290 *m*/*z* for positive and 556.0020 *m*/*z* for negative ion mode. MS/MS fragmentation was conducted by collision-induced dissociation of the five most abundant ions per MS scan with collision energies between 14 and 110 eV ,depending on the mass and charge of the parent ions. MS2 spectra were recorded at a scan rate of 10 Hz (for both C18 and HILIC chromatography).

Raw data were analyzed using the MetaboScape 4.0 software for peak picking and feature detections with parameters listed in Table S4. The lists of features were obtained using the MetaboScape software with a cutoff of retention time (0.3 min ≤ RT ≤ 28 min). Metabolites were further annotated by matching the retention time, MS and MS/MS fragmentation patterns with the in-house library of 600 metabolites as pure chemical standards, commercial libraries including LipidBlast (with 14048 metabolites), Bruker MetaboBase (482025 metabolites), and Bruker HMDB Metabolite Library (824 metabolites). Features were putatively identified by matching MS/MS fragmentation patterns as well as exact masses to an in-house library, open-source MS/MS libraries such as ECMDB,^69^ HMDB,^70^ GNPS,^71^ or theoretical MS/MS fragmentation patterns from MetFrag.^72^

### Time-Lapse Microscopy

Bacterial cultures (OD_600_ = 0.01) were prepared with or without antibiotic treatment in iron-limited MHB broth as mentioned above. For time-lapse imaging, the 96-well plate was incubated at 37 °C. Image acquisition was carried out with 30 min intervals over 24 h. The time-lapse images were collected on a Nikon-Ti2 Eclipse microscope at differential interference contrast view equipped with a 100× oil-immersion objective lens.

### Statistic Analysis, Bioinformatics, and Data Availability

Statistic analysis of metabolomics data was calculated by R packages-stats (version 3.6.3). *t* test correction was obtained by false discovery rate correction using the Benjamini-Hochberg method.^68^ The threshold for significant features is a corrected *p*-value (false discovery rate, FDR) < 0.05 and an absolute value of fold change >1.5. Lists of significantly regulated metabolites were further annotated with pathway information from the EcoCyc database^43^ and MetaboAnalyst.^73^ For visualization of data, the *R* packages pheatmap (version 1.0.12), ggplot2 (version 3.3.0), limma (version 3.42.2), EnhancedVolcano (version 1.4.0), RDAVIDWebService (version 4.0.3),^33^ the software Cytoscape,^74^ and the add-in Enrichment Map^75^ were used. All transcriptome data are publicly available under https://www.ncbi.nlm.nih.gov/geo/query/acc.cgi under the GEO accession number GSE197880. All metabolomics data are publicly available under https://massive.ucsd.edu/ProteoSAFe/static/massive.jsp with the MassIVE identifier MSV000088837, MSV000088863, MSV000088864, and MSV000088865.

## Supporting Information Available

Supplementary figures on antibacterial activity with iron uptake-deficient strains, antibacterial activity in the presence of polyamines, multidimensional scaling plots for metabolome data, and enrichment analysis and supplementary tables on regulated pathways, used primers, metabolomics search parameters, and OD-CFU correlations (PDF)
Data sets for download as separate files include lists of differentially expressed genes (sheet 1), enriched gene terms (sheet 2), and all identified metabolites and their relative abundances from untargeted metabolomics experiments (sheet 3) (XLSX)
