# Supplementary material for: Molecular Signatures of the Eagle Effect Induced by the Artificial Siderophore Conjugate LP-600 in E. coli
Source: ACS Infect Dis. 2023 Feb 10;9(3):567–81. doi: 10.1021/acsinfecdis.2c00567 (PMC10012262; doi:10.1021/acsinfecdis.2c00567)
Supplement: Supplementary file 1 — id2c00567_si_001.pdf [file id2c00567_si_001.pdf]

SUPPORTING INFORMATION

Molecular signatures of the Eagle effect induced by the artificial siderophore conjugate LP-600 in *E. coli*

Yi-Hui Lai,[1] *Raimo Franke,*[1] *Lukas Pinkert,*[1] *Heike Overwin*[1] *and Mark Brönstrup*[1,2,3*]

[1] Department of Chemical Biology Helmholtz Centre for Infection Research, Inhoffenstrasse 7, 38124 Braunschweig, Germany

[2] German Center for Infection Research (DZIF), Site Hannover-Braunschweig, 38124 Braunschweig, Germany

[3] Center of Biomolecular Drug Research (BMWZ), Leibniz University, 30159 Hannover, Germany

* mark.broenstrup@helmholtz-hzi.de

Table of Contents

Figure S1. Role of iron uptake for the antibacterial activity of LP-600 against *E. coli* 3

Figure S2. Multidimensional scaling (MDS) analysis of *E. coli* metabolomes recorded by UPLC-ESI-QToF 4

Figure S3. Functional enrichment analysis for differentially expressed genes shared between LH0.5 and LH1 comparisons 5

Figure S4. Networks of selected functional enrichment with significantly up-regulated (A) and down-regulated (B) genes in the exponential growth phase (LH0.5). 6

Figure S5. 7

Figure S6. Antibacterial activity of LP-600 against *E. coli* in the presence of polyamines 8

Table S1. Top 100 differentially regulated pathways. 9

Table S2. List of primers for lambda red-mediated gene deletion. 11

Table S3. List of primers for colony PCR. 12

Table S4. List of parameters applied in Metaboscape. 13

Table S5. Correlation of OD and CFUs 16

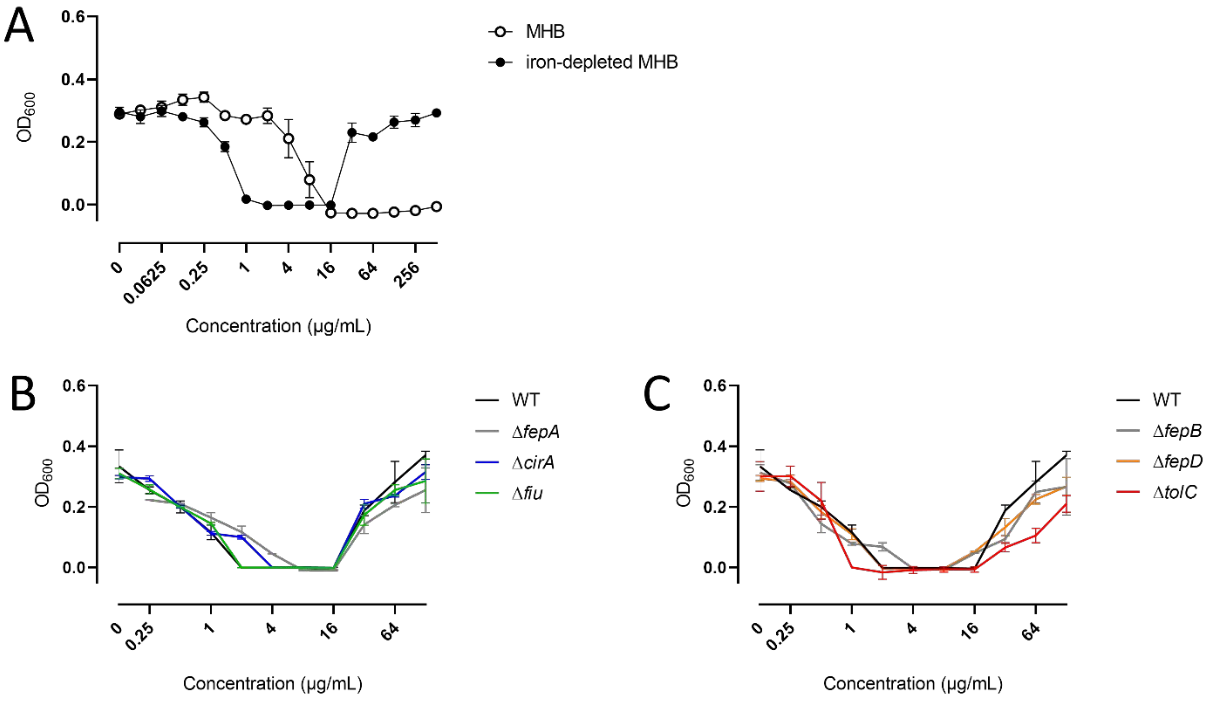

Figure S1. Role of iron uptake for the antibacterial activity of LP-600 against *E. coli*. (A) *E. coli* BW25113 was treated with LP-600 with iron-depleted or standard Mueller Hinton broth (MHB) for 24 hours, followed by OD_{600} measurement. Strains deficient of genes *fepA, cirA*, and *fiu* (B), or *fepB, fepD*, and *tolC* (C) were treated with LP-600 for 24 hours in iron-depleted MHB, followed by OD_{600} measurements. Representative results of n = 3.

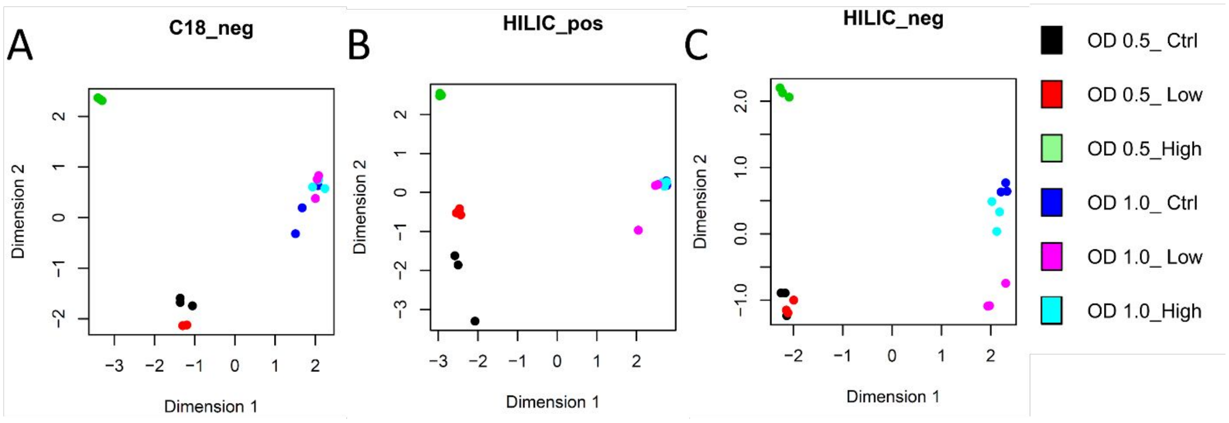

Figure S2. Multidimensional scaling (MDS) analysis of *E. coli* metabolomes recorded by UPLC-ESI-QToF. (A-C) C18 or HILIC columns were used for analyte separations in positive or negative mode. Results obtained with a C18 column in positive mode are depicted in Figure 3. The cultures were treated with DMSO (Ctrl), low and high-concentrations of LP-600 during the mid-exponential phase (OD_{600} = 0.5) or stationary phase (OD_{600} = 1.0). Log-CPM (log counts per million) values among samples were applied in MDS analysis with the R package limma to project Euclidean distances between samples to x- and y-axis.

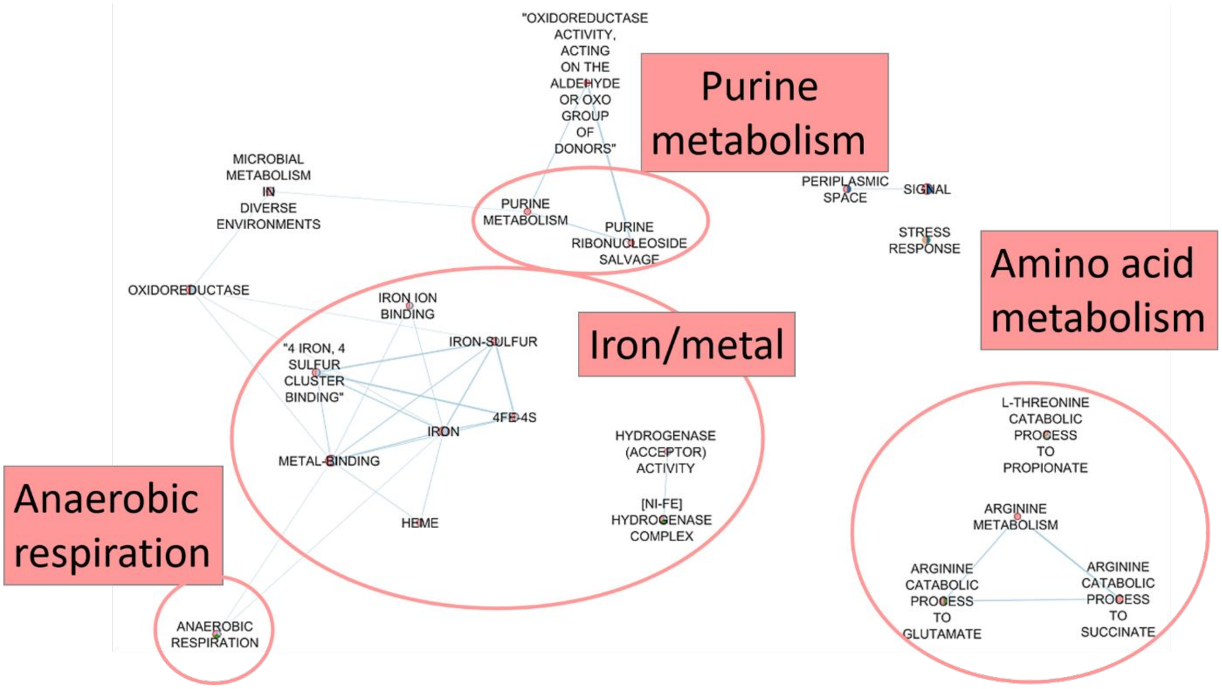

Figure S3. Functional enrichment analysis for differentially expressed genes shared between LH0.5 and LH1 comparisons. Networks of functionally enriched terms associated with significantly regulated genes under both LH0.5 and LH1 conditions are displayed. Each node represents an enrichment category and is labeled with its correlating annotation. The line between two nodes represents genes overlapping among them. The overall number of categories is 22.

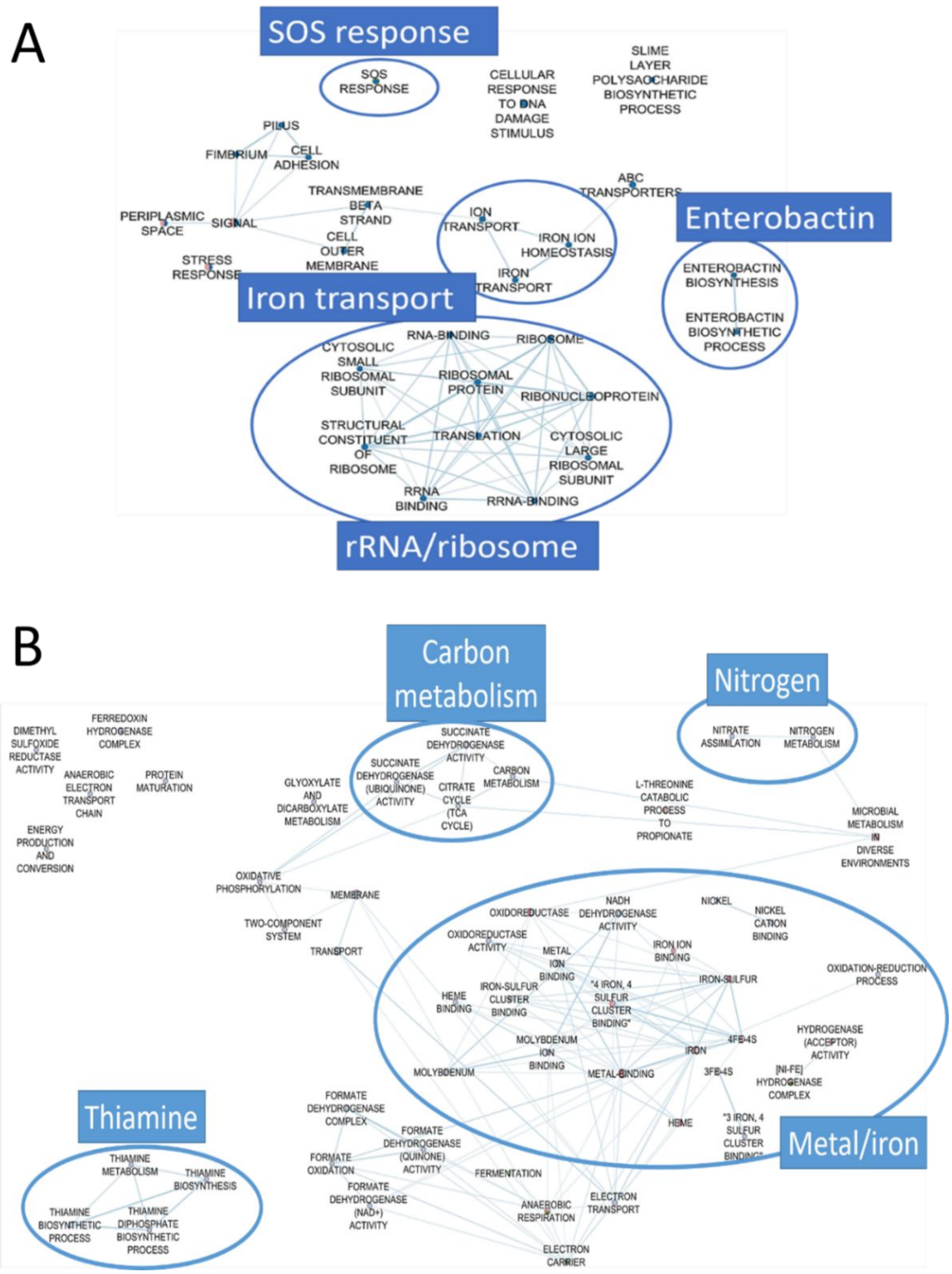

Figure S41. Networks of selected functional enrichment with significantly up-regulated (A) and down-regulated (B) genes in the exponential growth phase (LH0.5). Each node represents an enrichment category and is labeled with the annotation. The line between two nodes represents genes overlapping in two nodes. LH0.5: Comparison of high-concentration over low-concentration treatment with LP-600 at OD_{600} = 0.5.

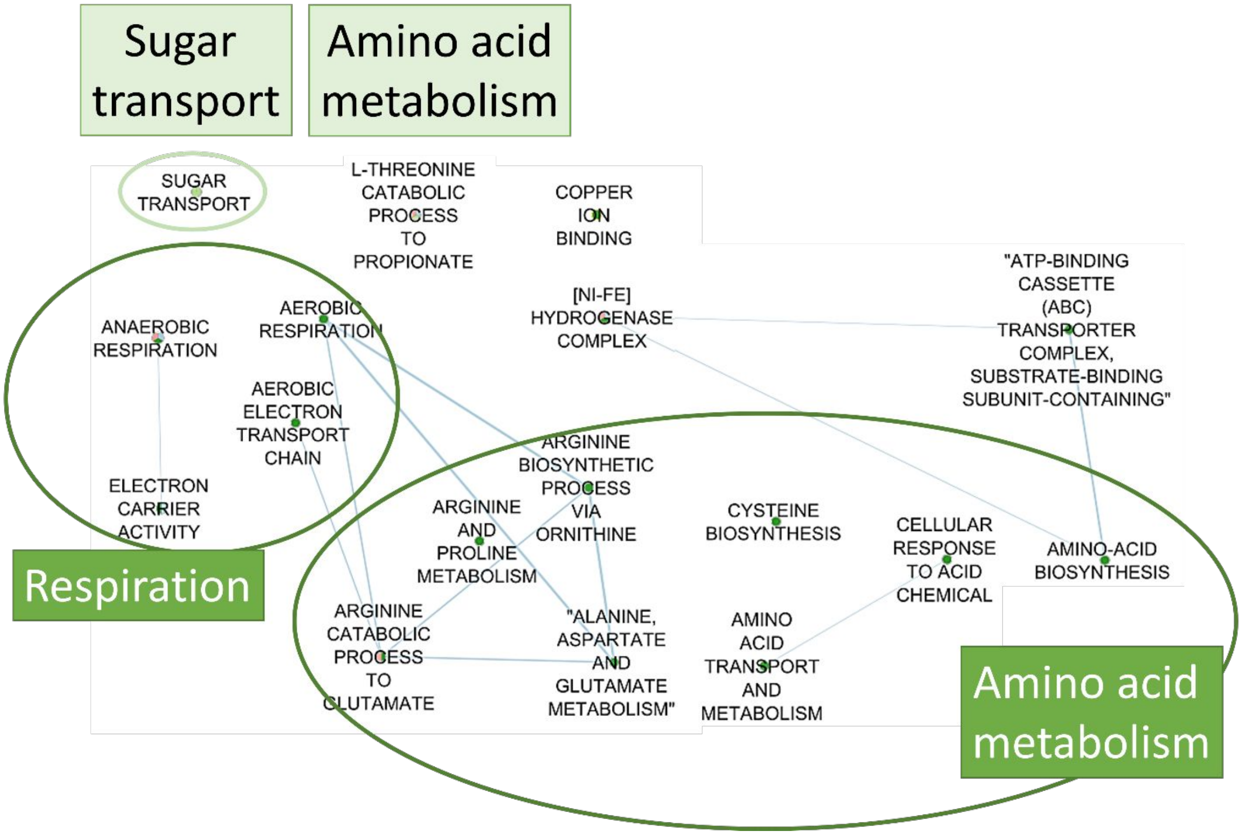

Figure S5. Networks of selected functional enrichment with significantly regulated genes in the stationary phase (LH1)**.** Functional enrichment of networks with differentially expressed genes in LH1. Each node represents an enrichment category and is labeled with the annotation. The line between two nodes represents genes overlapping in two nodes. Nodes and clusters are labeled in dark green for terms with up-regulation genes, whereas light green represents a term with down-regulated genes.

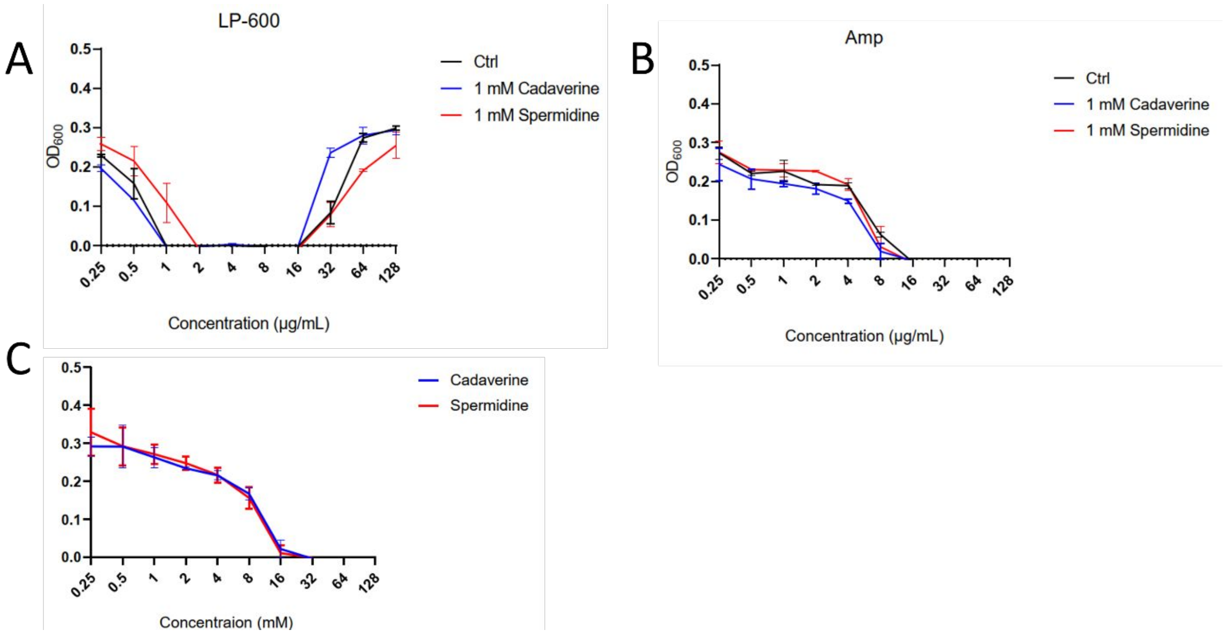

Figure S6. Antibacterial activity of LP-600 against *E. coli* in the presence of polyamines**.** (A-B) *E. coli* BW25113 was treated with LP-600 (A) or ampicillin (B) with iron-depleted MHB for 24 hours in the presence of 1 mM cadaverine or spermidine, followed by OD_{600} measurement. (C) Antibacterial activity of cadaverine or spermidine against *E. coli*. Representative results of n = 3.

Table S1. Top 100 differentially regulated pathways.

Pathway	DPPS*	Pathway	DPPS*	Pathway	DPPS*	Pathway	DPPS*
Cadaverine biosynthesis	9.0	Glycerol degradation V	6.4	Mixed acid fermentation	5.4	L-cysteine biosynthesis I	4.8
Biotin biosynthesis from 8-amino-7-oxononanoate I	8.9	Enterobactin biosynthesis	6.3	Superpathway of thiamine diphosphate biosynthesis I	5.4	Glyoxylate cycle	4.8
Lipoate biosynthesis and incorporation II	8.7	Heme- biosynthesis II (oxygen-independent)	6.3	L-cysteine biosynthesis VII (from sulfo-L-cysteine)	5.3	CDP-diacylglycerol biosynthesis I	4.8
4-amino-2-methyl-5-diphosphomethylpyrimidine biosynthesis I	8.7	Methylphosphonate degradation I	6.3	Hydrogen to fumarate electron transfer	5.3	2-methylcitrate cycle I	4.8
Lipoate biosynthesis and incorporation I	8.7	Ethanol degradation I	6.2	Citrate degradation	5.3	Fatty acid β-oxidation I (generic)	4.8
Hydrogen to trimethylamine N-oxide electron transfer	8.6	L-threonine degradation II	6.2	Biotin biosynthesis I	5.2	Thiazole component of thiamine diphosphate biosynthesis I	4.8
Glutathionylspermidine biosynthesis	8.0	2,3-dihydroxybenzoate biosynthesis	6.0	Superpathway of methylglyoxal degradation	5.2	Superpathway of arginine and polyamine biosynthesis	4.8
Arsenate detoxification II (glutaredoxin)	8.0	NADH to dimethyl sulfoxide electron transfer	6.0	Superpathway of heme biosynthesis from uroporphyrinogen-III	5.2	Cinnamate and 3-hydroxycinnamate degradation to 2-hydroxypentadienoate	4.8
NADH to trimethylamine-oxide electron transfer	7.9	L-arginine degradation III (arginine decarboxylase/agmatinase pathway)	5.8	(Aminomethyl)phosphonate degradation	5.1	Nitrate reduction III (dissimilatory)	4.7
Superpathway of L-aspartate and L-asparagine biosynthesis	7.7	Putrescine biosynthesis I	5.8	Molybdopterin biosynthesis	5.1	Superpathway of glyoxylate bypass and TCA	4.7
Superpathway of L-asparagine biosynthesis	7.7	Glutathione biosynthesis	5.7	3-dehydroquinate biosynthesis I	5.1	Formate to nitrite electron transfer	4.7
L-asparagine degradation I	7.7	Superpathway of purine deoxyribonucleosides degradation	5.7	Pyruvate decarboxylation to acetyl coa I	5.1	TCA cycle I (prokaryotic)	4.7
β-alanine biosynthesis III	7.7	Nitrate reduction VIII (dissimilatory)	5.7	Formate to dimethyl sulfoxide electron transfer	5.1	Superpathway of polyamine biosynthesis I	4.7
L-asparagine biosynthesis I	7.7	Nitrate reduction viiib (dissimilatory)	5.7	2-oxoglutarate decarboxylation to succinyl-coa	5.0	L-arginine degradation II (AST pathway)	4.6
L-asparagine biosynthesis II	7.7	Glutathione-glutaredoxin redox reactions	5.7	L-threonine degradation I	5.0	2-carboxy-1,4-naphthoquinol biosynthesis	4.5
L-aspartate biosynthesis	7.7	Superpathway of acetate utilization and formation	5.6	Ethanolamine utilization	5.0	NAD de novo biosynthesis I (from aspartate)	4.5
L-glutamate degradation II	7.7	Fatty acid biosynthesis initiation (type II)	5.5	Acyl carrier protein metabolism	5.0	NADH to cytochrome <i>bd</i> oxidase electron transfer I	4.5
Arginine dependent acid resistance	7.6	D-malate degradation	5.5	Acetate and ATP formation from acetyl-coa I	4.9	NADH to cytochrome <i>bd</i> oxidase electron transfer II	4.5

Formate to trimethylamine-oxide electron transfer	7.3	UDP-α-D-glucuronate biosynthesis (from UDP-glucose)	5.5	Oleate β-oxidation	4.9	D-gulosides conversion to D-glucosides	4.5
Hydrogen to dimethyl sulfoxide electron transfer	6.8	NAD phosphorylation and dephosphorylation	5.5	Acetoacetate degradation (to acetyl coa)	4.9	L-phenylalanine biosynthesis I	4.5
Acetate conversion to acetyl-coa	6.8	Ethylene glycol degradation	5.5	Fatty acid biosynthesis initiation II	4.9	Adenosine ribonucleotides <i>de novo</i> biosynthesis	4.5
Purine deoxyribonucleosides degradation I	6.7	D-sorbitol degradation II	5.5	L-threonine degradation IV	4.9	Superpathway of glycolysis, pyruvate dehydrogenase, TCA, and glyoxylate bypass	4.5
Methylglyoxal degradation I	6.5	Mannitol degradation I	5.5	Glycolate and glyoxylate degradation II	4.9	Superpathway of glycol metabolism and degradation	4.5
Formaldehyde oxidation II (glutathione-dependent)	6.5	L-lactaldehyde degradation (anaerobic)	5.5	Superpathway of fatty acid biosynthesis initiation (*E. coli*)	4.8	L-homoserine biosynthesis	4.4
Aminopropylcadaverine biosynthesis	6.4	L-galactonate degradation	5.5	L-arginine biosynthesis I (via L-ornithine)	4.8	Superpathway of L-threonine biosynthesis	4.4

*DPPS: Differential Pathway Perturbation Score.

Table S2. List of primers for lambda red-mediated gene deletion.

**Primer**	**Sequence**
fepA-K-F	CCGCATCCGGCATGAACGACGCGCACTTTGTCAACAATCTGACGTTAGCAGTGTAGGCTGGAGCTGCTTC
fepA-K-R	CGACCATGCCCGACAGTTGCAATTCGTGGCAAAAATGCAGGAATAAAACAATGGGAATTAGCCATGGTCC
K-fepB-F	CGCAGGTGACAGCGTCCGACAGTTAATGCTTAAAACAGCGCCTTAAGCCTGTGTAGGCTGGAGCTGCTTC
K-fepB-R	AATTTGTCATTACGCCCTTAACCTTATTAATAACAGGAAGCTGATTTGTGATGGGAATTAGCCATGGTCC
K-fepD-F	GGTGATGAGTAATCGGCGAGAGACGTAAATCATGCACCACCTCGCGTTTTGTGTAGGCTGGAGCTGCTTC
K-fepD-R	AAATAAGATCGATAACGATAATTAATTTCATTATCATGGAAGTTCGTATGATGGGAATTAGCCATGGTCC
K-fecD-F	ACCGTCAGATTTTCAGTTCGTAAAGTCATTTATCGCATTCTCACAAGCAAGTGTAGGCTGGAGCTGCTTC
K-fecD-R	GCGCTGATTGGCAGCCCTTGCTTTGTCTGGCTTGTGAGGAGGCGAGGATGATGGGAATTAGCCATGGTCC
K-cirA-F	GCAGTATTTACTGAAGTGAAAGTCCGCCCGTCGCCGGGCATCTTCTCAGTGTAGGCTGGAGCTGCTTC
K-cirA-R	TGTGAGCGATAACCCATTTTATTTTCGTAGTTACCTCATGGAGATATGGAATGGGAATTAGCCATGGTCC
K-fiu-F	GTACATCATACAATTTCTCCAAAAAGTGGGGCCTGCGCCCCACATCTGAAGTGTAGGCTGGAGCTGCTTC
K-fiu-R	TTTCTCGTGGCAGTGAAAATTTCAACATATAAGAAAAAGTCACCTGCAAAATGGGAATTAGCCATGGTCC

Table S3. List of primers for colony PCR.

Primer	Sequence
fepA-C-F	CCGCATCCGG CATGAACGAC GCGCA
fepA-C-R	CGACCATGCCCGACAGTTGCAATTC
C-fepB-F	CGCAGGTGACAGCGTCCGACAGTTA
C-fepB-R	AATTTGTCATTACGCCCTTAACCTT
C-fepD-F	GGTGATGAGTAATCGGCGAGAGACG
C-fepD-R	AAATAAGATCGATAACGATAATTAA
C-cirA-F	GCAGTATTTA CTGAAGTGAA AGTCC
C-cirA-R	TGTGAGCGATAACCCATTTTATTTT
C-fiu-F	GTACATCATA CAATTTCTCC AAAAA
C-fiu-R	TTTCTCGTGGCAGTGAAAATTTCA

Table S4. List of parameters applied in Metaboscape.

Parameters	Positive	Negative
ferraWorkflow_minCorrelation	0.8	0.8
ferraWorkflow_lockMass	622.0290	556. 0020
ferraWorkflow_GroupFeatures_rtDelta	10	10
ferraWorkflow_chargeMax	3	3
ferraWorkflow_rtMaxInSeconds	1680	1680
ferraWorkflow_ForeachAnalysisMsms_MsmsExtractionWorkflow_ ConsolidateMsmsPeaklists_method	average	average
msmsExtractionCompassResult_fillNonDeconvolutedValue	0	0
ferraWorkflow_substanceClass	small molecules	small molecules
ferraWorkflow_rtMinInSeconds	12	12
ferraWorkflow_ForeachAnalysis_FeatureFinder_ClusterDeisotoping_ featureIntervalMethod	FWHM	FWHM
ferraWorkflow_seedIntensityThreshold	500	500
ferraWorkflow_enableLockMass	true	true
ferraWorkflow_useIsotopePatternCoverage	false	false
ferraWorkflow_ForeachAnalysisMsms_MsmsExtractionWorkflow_ MsmsDeisotoping_relativeAbundanceThreshold	0.005	0.005
ferraWorkflow_targetedExtractionMinClusterSize	3	3
ferraWorkflow_maxClusterOverlap	0.1	0.1
ferraWorkflow_ForeachAnalysisMsms_MsmsExtractionWorkflow_	true	true

ConsolidateMsmsPeaklists_groupByCollisionEnergy		
ferraWorkflow_mzMin	75	75
ferraWorkflow_ForeachAnalysis_FeatureFinder_ClusterDeisotoping_ areaCalculationScale	2	2
ferraWorkflow_minExistFraction	0.55	0.55
ferraWorkflow_CreateRecursiveTargets_threshold	3	3
ferraWorkflow_ForeachAnalysisMsms_MsmsExtraction Workflow_MsmsDeisotoping_proteomics	true	true
msmsExtractionCompassResult_fillStrategy	topN	topN
ferraWorkflow_uffMinSeedClusterSize	7	7
ferraWorkflow_maxIsotopePatternError	0.2	0.2
ferraWorkflow_CreateBatchFeatures_minGroupSize	3	3
ferraWorkflow_minCorrelatedFraction	0.55	0.55
ferraWorkflow_mzMax	1000	1000
ferraWorkflow_areaIntensity	false	false
ferraWorkflow_enableMsmsExtraction	true	true
ferraWorkflow_minNumClusters	1	1
ferraWorkflow_uffMinClusterSize	2	2
processingWorkflowId	Ferra3d	Ferra3d
polarity	positive	negative
Deconvolution.eicCorrelation	0.8	0.8
Persist Only ConsensusIsotope Pattern	false	false
Deconvolution.primaryIon	[M+H]+	[M-H]-

Deconvolution.seedIons	[M+Na]+, [M+K]+	[M+Cl]-
Deconvolution.commonIons	[M-H2O+H]+, [2M+H]+, [M+NH4]+, [M-CO2+H]+	[M-H-H2O]-
Sample Group Filter Type	percentage	percentage
Sample Group Presence Filter Value	100	100
Nupf Time Stamp	1.60215E+12	1.6E+12
Nupf Workflow Version	3.4	3.4

Table S5. Correlation of OD and CFUs

conc. [µg/ml]	OD_{600nm}	CFU/well	Pearson´s r
64	0.1447	2.20E+08	0.96
64	0.1575	2.00E+08	
64	0.0928	5.74E+07	
32	0.2286	6.49E+08	0.93
32	0.2001	2.57E+08	
32	0.1713	1.87E+08	
16	0.0221	5.27E+07	1.00
16	0.0385	4.41E+07	
16	0.2114	4.50E+08	
8	0.0023	1.00E+01	0.95
8	0.001	0.00E+00	
8	0.0014	0.00E+00	
4	0.0127	9.90E+07	0.99
4	0.1591	5.13E+08	
4	-0.0013	1.00E+01	
2	0.2149	3.50E+08	1.00
2	0.0014	9.50E+01	
2	0.0019	3.75E+01	
1	0.1543	2.17E+08	0.98
1	0.1647	4.27E+08	
1	0.1509	2.02E+08	
0.5	0.2137	5.24E+08	0.99
0.5	0.1544	2.12E+08	
0.5	0.1549	1.55E+08	
0.25	0.2745	7.24E+08	0.91
0.25	0.1998	4.75E+08	
0.25	0.1944	2.50E+08	

Overall Pearson´s r = 0.87
